# Efficacy and Cost-Effectiveness of Phenotyping for Rice Resistance and Tolerance to Planthoppers

**DOI:** 10.3390/insects12100847

**Published:** 2021-09-22

**Authors:** Finbarr G. Horgan, Enrique A. Mundaca, Reyeul Quintana, Alberto I. Naredo, Maria Liberty P. Almazan, Carmencita C. Bernal

**Affiliations:** 1 EcoLaVerna Integral Restoration Ecology, Bridestown, Kildinan, T56 P499 County Cork, Ireland; 2 Escuela de Agronomía, Facultad de Ciencias Agrarias y Forestales, Universidad Católica del Maule, Casilla 7-D, Curicó, Chile; emundaca@gmail.com; 3 International Rice Research Institute, DAPO Box 7777, Metro Manila 1301, Philippines; r.quintana@irri.org (R.Q.); a.naredo@irri.org (A.I.N.); m.l.almazan@irri.org (M.L.P.A.); c.bernal@irri.org (C.C.B.)

**Keywords:** breeding, *BPH32*, days-to-wilt, honeydew, host plant resistance, leafhoppers, *Nilaparvata*, oviposition

## Abstract

**Simple Summary:**

Rice in Asia is damaged by a range of sap-sucking planthoppers. For the last 50 years, the main focus for integrated management of these insects was to develop resistant rice varieties. A single, bulk phenotyping test, known as the standard seedling seedbox test (SSST) was used to inform the majority of genetics and breeding programs for planthopper-resistant rice. However, there has been much debate over the efficacy of this test. We compared results from the SSST against those from a range of other phenotyping tests to assess how antibiosis and tolerance (the ability of rice to compensate for damage) affect test results, and how phenotyping might be improved to capture information regarding plant traits that are otherwise overlooked by the SSST. Based on the efficacy and costs of different tests, we recommend that breeding programs adopt a modified seedling seedbox test (MSST) when phenotyping 100s of rice lines but that insect performance tests that assess relative changes in planthopper biomass could be adopted when phenotyping fewer lines (e.g., <100 lines). The days-to-wilt (DTW) test was found to be particularly effective in segregating resistant/tolerant lines from susceptible lines.

**Abstract:**

The standard seedling seedbox test (SSST) is the most prevalent phenotyping test in research on the genetics and breeding of planthopper-resistant rice. Using 16 rice lines that included plants susceptible, resistant and tolerant to the brown planthopper (*Nilaparvata lugens*), we compared the SSST to modified seedling seedbox tests (MSSTs) and the days-to-wilt (DTW) test. We also conducted a series of performance tests to assess nymph survival and development; adult longevity and egg-laying; egg survival; honeydew production; and plant weight loss. We also assessed the relative costs of the different phenotyping tests to better recommend test protocols that are suitable for high-throughput phenotyping. The SSST was found to be highly robust but fails to identify late-stage resistance; tolerance; or ovicidal responses. MSSTs improved phenotyping by identifying plants with low damage from planthoppers at later growth stages. Herbivore performance tests such as population or biomass build-up tests reduce space requirements and reduce setup and evaluation costs compared with bulk tests. They can also facilitate the assessment of plant tolerance; albeit with added costs. The DTW test most clearly segregates resistant and susceptible plants, thereby facilitating gene discovery and marker-assisted selection. We recommend that bulk testing be improved by switching from the SSST to a suitable MSST and that donor variety and pre-release lines be assessed for the nature of rice–planthopper interactions using biomass build-up tests—including the DTW test.

## 1. Introduction

Among the most damaging insect pests of rice in Asia are a range of planthoppers (Hemiptera: Delphacidae) and leafhoppers (Hemiptera: Cicadellidae). These can cause direct mechanical damage to rice by feeding on plant phloem and xylem or cause indirect damage by transmitting rice viruses [1,2,3]. Planthoppers in particular have become widespread throughout Asia and frequently exceed economic threshold densities to cause extensive losses to rice yields [4,5]. Outbreaks have been linked to an overuse of chemical fertilizers and insecticides [6,7]. Convergent weather systems may also facilitate the movement of planthopper populations to rice fields in northeast Asia where sudden increases in population density can overwhelm natural enemies [1,8]. Therefore, the management of these planthoppers and leafhoppers has been based on reducing agrochemical use, avoiding or eliminating insecticides that stimulate planthopper feeding and development (i.e., resurgence-causing), and restoring rice ecosystems to improve the efficiency of natural enemies such as egg parasitoids and predatory hemipterans [7,9,10]. One principal activity in the restoration of rice ecosystems for the management of planthoppers and leafhoppers is to ensure that susceptible rice varieties are avoided and that resistant varieties are developed and deployed to farmers’ fields [11]. 

In recent decades, there has been a growing interest in using molecular tools during the breeding of rice varieties with anti-herbivore resistance [11,12,13]. By applying marker-assisted selection, researchers have successfully tagged and transferred one or more resistance genes from donor rice varieties to a range of high-yielding varieties (reviewed by Horgan et al. 2018 [14]). These include varieties with the *BPH14*, *BPH15*, and/or *BPH32* genes either as monolocus or multiloci (pyramided) resistance [14,15,16,17]. The mechanisms underlying resistance in rice include physical/mechanical factors such as plant hairs, thick or waxy cuticles, or high-silicon plants tissues, and biochemical factors that include constitutive and induced defense compounds such as sterols, flavonoids or complex volatiles that act as repellents, anti-feedants, anti-digestives or toxins [11]. These defenses are often polygenetic in nature and major resistance genes will sometimes interact with each other or with the plant’s genetic background to express novel resistance traits [7,11]. Therefore, resistance in donor varieties is typically complex and many of the donor varieties that are most commonly used in breeding programs have multiple major resistance genes as well as quantitative resistance traits [11]. 

Despite the large number of major anti-planthopper or anti-leafhopper genes that have been identified in rice (currently about 60 genes [11,12,16]), the resistance mechanisms associated with these genes are often poorly understood. However, the functioning of some of the major resistance genes was elucidated. For example, the *BPH32* gene encodes a protein with a signal peptide and SCR-domain that inhibits feeding by binding to planthopper glycoproteins or tissues [15]. Varieties with the *Bph3* gene have relatively high concentrations of schaftocides that produce anti-feeding effects. The ovicidal response in *japonica* rice varieties is associated with the *Ovc* gene and several quantitative trait loci; these plants respond to planthopper egg insertion into the plant by producing a necrotic lesion around the egg cluster that fills with benzyl benzoate to kill the eggs. Much of the response to planthopper and leafhopper feeding in rice involves activation of the salicylic acid defense pathway with final biosynthesis of defense volatiles and anti-feeding enzymes [11,12]. Therefore, rice defenses against planthoppers and leafhoppers can be divided into two major response groups, these are antixenosis defenses that reduce settling and oviposition (often referred to as preference responses) and antibiosis defenses that reduce herbivore performance (e.g., growth, development, egg hatching). Ultimately, both antixenosis and antibiosis reduce damage to the rice plant; however, the degree of damage from herbivores also depends on the plant’s tolerance to herbivory (i.e., the ability to compensate for resources lost through insect feeding).

**Figure 1:**
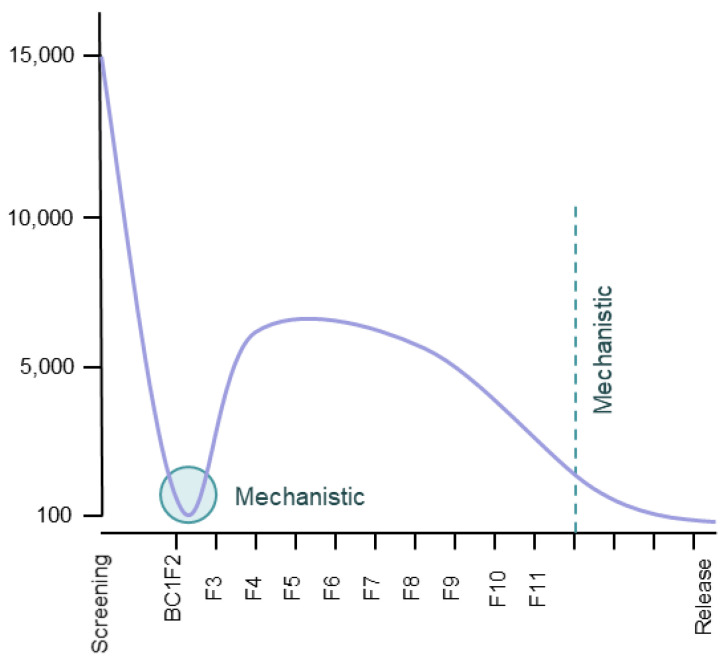
Typical numbers of rice plants at successive stages of IRRI’s breeding pipeline (data from 2009–2015). Initial screening of varieties and landraces identified a limited number of genotypes with resistance to planthoppers and leafhoppers. These were then integrated into the breeding program together with genotypes possessing other desirable traits (<100 genotypes). Crossing increases the number of plants that require screening. At an estimated cost of $1 per line, bulk tests are recommended for screening and early generations such as back cross F2 (BC1F2) and F3-F11 generations. Mechanistic research is recommended for donor varieties/landraces, at post-F11 generations and at pre-release (graph and cost estimates are based on unpublished data from Horgan and Almazan).

Although resistant rice varieties have been released to rice farmers, much of the reported evidence for the success of these varieties in reducing herbivore damage still comes from simple greenhouse or laboratory trials using bulk or rapid evaluation tests. In this paper, we consider a bulk test as any test that simultaneously evaluates several rice varieties (i.e., the experimental unit incorporates several rice phenotypes). We contrast bulk tests with tests designed to evaluate herbivore performance, where the experimental unit includes only a single rice phenotype, usually planted in a pot. Such “performance tests” can often be rapidly evaluated by scoring plant damage, or they can be evaluated using detailed measures related to herbivore fitness (e.g., survival, insect weights, development times, sex ratios). We refer to tests that rate damage as “rapid tests”; (bulk tests are normally rapid tests because they are evaluated by rating plant damage). The discovery of resistance genes and the development of rice varieties with resistance to planthoppers and leafhoppers will often depend entirely upon rating plant damage from bulk tests such as the standard seedling seedbox test (SSST) and the modified seedling seedbox test (MSST) [11] or other rapid tests [18,19,20,21]. This is mainly because large quantities of breeding materials must be screened during the development of new varieties, and the costs per individual line need to be maintained at a minimum. For example, Figure 1 shows the approximate number of rice lines evaluated for planthopper and leafhopper resistance at the International Rice Research Institute (IRRI) in the Philippines before the release of new varieties each year; rapid tests are essential to screen such large numbers of lines. However, because of tradeoffs between the cost and reliability of tests, rapid phenotyping tests are limited in terms of the information they can provide, and bulk tests, in particular, have been criticized for focusing only on antibiosis-based resistance in rice seedlings [18,20,21,22,23,24].

Horgan et al. [19] explain that rapid tests, such as the SSST, will only give information on relative damage to test plants and not on relative plant resistance or tolerance. However, these authors also suggest that the SSST could be modified to improve information capture. For example, relative resistance and relative tolerance could be assessed by recording insect densities on plants, and by incorporating non-infested controls into test protocols, respectively. Without such measures, it is not possible to determine what it is that bulk test results are actually capturing (i.e., antixenosis, antibiosis, tolerance). Any true measure of resistance will require a focus on changes in insect fitness (survival × reproduction) [25]. Meanwhile, evaluations of tolerance require accurate measures of plant responses to quantified feeding by insect herbivores at two or more densities (i.e., changes in biomass, physiological changes, changes to color or chlorophyll content, or changes in yield [26,27,28]). A large number of methods aimed at more detailed phenotyping for rice resistance are available [29,30]; however, few studies have compared the efficiency of phenotyping methods in distinguishing resistance and tolerance in rice plants [12,24,28], and to our knowledge, no study has compared the relative costs of different phenotyping methods. Furthermore, many previous studies that report the results of different phenotyping tests using the same plant materials, have applied tests that cannot be directly compared to standard bulk tests (i.e., compared to the SSST these studies used differently aged plants, different densities of planthoppers, or different bioassay durations [21,31,32]). Although these studies do indicate some of the limitations of bulk tests, they cannot explain SSST or MSST results in terms of the nature of herbivore–plant interactions during the tests. 

This study aims to improve phenotyping during varietal development by recommending an optimal array of tests that can better capture information on herbivore-rice interactions through the breeding pipeline in a cost-effective manner. The study does not consider field screening. Using a range of rice varieties for which interactions with the brown planthopper (BPH), *Nilaparvata lugens* (Stål), have been relatively well-documented, we assessed the efficiency and cost-effectiveness of three different bulk tests (the SSST and two MSSTs), and a further rapid-evaluation test (the days-to-wilt test [DTW]), to accurately detect resistance and/or tolerance to the planthopper in rice. The study had three main objectives. (1) to assess some of the biological factors (e.g., antibiosis or tolerance) and compare results from bulk tests with detailed performance tests, (2) to correlate the duration of bulk tests with development times in performance tests to assess the degree to which information pertinent to resistance breeding may be neglected or overlooked and (3) to assess the relative costs of phenotyping tests and determine how cost-benefits could be optimized to improve breeding for planthopper resistance. 

## 2. Materials and Methods

### 2.1. Rationale for Study Design

We assessed three bulk tests (the SSST [33] and two versions of the MSST as described by Velusamy et al. (1986) [20]) and the DTW test, which is a prominent, rapid phenotyping test, for their capacities to distinguish resistant and tolerant rice plants from susceptible/low tolerance rice plants. Each of these tests is normally run for between 7 and 40 days and evaluated using the standard evaluation system (SES) that rates damage to the plants on a scale from 0 to 9 (see footnote in Table 1). The days-to-wilt (DTW) test normally runs for 30 to 60 days; it can be evaluated using the SES, or by simply noting the time in days at which plants wilt due to planthopper damage. Although further data can be gleaned from the DTW test (i.e., insect population build-up, insect development, biomass accumulation of insects), the DTW test is normally run as a rapidly evaluated test [34,35]. To better distinguish the many tests that we conducted, we use subscripts to indicate the age of plants at infestation and the density of insects per plant as used in the tests. We also indicate whether insects were nymphs or adults by adding the subscript “n”, “f”, or “f/m” to indicate nymphs, adult females or adult male–female pairs, respectively; we use “ḟ” to indicate gravid females (e.g., SSST_7.8n_ indicates that plants were infested at 7 days after sowing [DAS] by eight nymphs). Nymphs were always ≤2nd instars. Table 1 presents a summary of the test conditions, recorded parameters and the location of test results.

**Figure 2:**
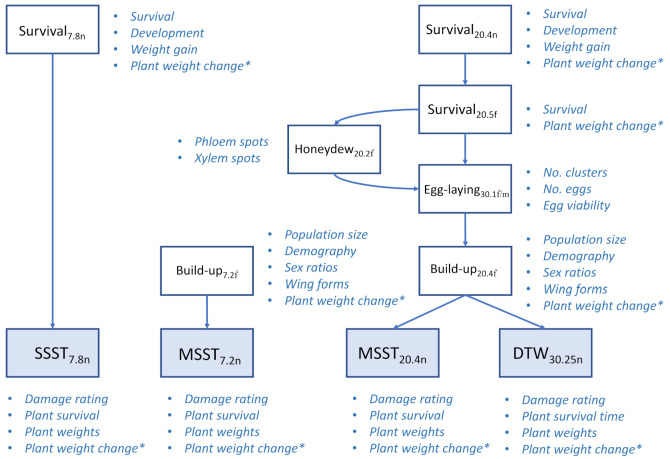
Schematic showing the parameters collected from bulk (SSST and MSSTs) and rapid phenotypic evaluations (DTW test) for plant damage (shaded rectangles) and their relation to performance tests (open rectangles). Performance tests assess resistance (based upon measures of insect fitness) and tolerance (parameters indicated by asterisks). Subscripts indicate plant age at infestation and insect density per plant. See Table 1 for further details related to each test.

We assessed the SSST, MSSTs and DTW test against the performance tests. This allowed us to break down insect responses to the plant materials into quantifiable measures (Figure 2). Therefore, the results for plant parameters from the SSST_7.8n_ were compared against plant and herbivore parameters from a corresponding performance test. The performance test, therefore, used plants at seven DAS, and was infested with eight planthopper nymphs per plant (Figure 2). Similarly, we assessed the results of the MSST_7.2n_ against a performance test that used seven DAS seedlings infested with two planthoppers. However, because the MSST_7.2n_ is designed to allow F1 populations to develop, we ensured that F1 populations caused the final damage to the rice plants in our performance test by infesting the seedlings with adults and quantifying the build-up (population and accumulated biomass) of the resulting planthopper populations (i.e., MSST_7.2__ḟ_: Figure 2). 

The MSST_20.4n_ was originally designed to also allow populations to develop to lay eggs, oviposit and cause final damage as an F1 population (referred to as “field resistance” [20]). The age of plants and densities of planthoppers used in the DTW test can also be adjusted to allow F1 populations to develop and eventually damage or kill plants. To ensure development to an F1 population, we used plants at 30 DAS infested at a moderate density of 25 nymphs in our DTW test. Under field conditions, eggs and early instar nymphs of the brown planthopper can occur on plants across a range of ages, and egg-laying on plants at 30 DAS or older can occur where F1 planthoppers move to new plants in infested fields [9,27]. We compared the results from the MSST_20.4n_ and DTW_30.25n_ tests against a range of planthopper responses across the complete life-cycle of the insect. These included bioassays that assessed nymph survival and development, adult survival (longevity), adult honeydew production, egg-laying and egg survival and population build-up on plants of ≥20 DAS in the performance tests (Figure 2). 

All experiments except the honeydew tests were conducted under greenhouse conditions. The greenhouse had a natural light regime (12 h light and 12 h darkness) with temperatures fluctuating between 25 and 35 °C during the experiments. Relative humidity in the greenhouse fluctuated between a maximum of 90% after dawn and a minimum of 70% after midday. There was no incidence of rice disease or attack by other herbivores during the experiments. The honeydew test was conducted in an air-conditioned laboratory with relatively constant temperatures of ca 25 °C (humidity = 75%). For each experiment, all replicates were completed at the same time such that fluctuations in temperature and humidity were consistent across treatments. We used no pesticides or fertilizers during the experiments. Bioassays are each explained in the following sections with further details outlined in Table 1.

### 2.2. Plant Materials

We used a collection of accessions available from IRRI to represent as wide an array of resistance genes/sources as possible. This consisted of 16 accessions as follows (resistance genes are presented in square brackets): The rice varieties Taichung 65 (henceforth T65) and Taichung Native 1 (henceforth TN1) are closely related *Oryza sativa* spp. *japonica* varieties [36] that are highly susceptible to planthoppers and frequently used as standard checks. The only other *japonica* variety we used was Asominori, which displays an ovicidal response to *N. lugens* and the white-backed planthopper, *Sogatella furcifera* Horváth (possibly with the *Ovc* gene: [37]). The variety IR22 is an *O. sativa* spp. *indica* variety also with high susceptibility to the brown planthopper. Among the remaining *indica* materials we used IR40 [*bph2*] and IR65482-7-216-1-2-B (henceforth IR65482-7 [*BPH18*]) which form a clade with IR22, and are closely related to IR62 [*BPH32*] and IR65482-4-136-2-2 (henceforth IR65482-4 [*Bph10*]) [36]; the variety IR46 [*Bph1*] is closely related to the other IR varieties [38]. We also used TKM6 [*Bph1*], Mudgo [*Bph1*], ASD7 [*bph2*], Rathu Heenati [*Bph3*, *Bph17*], and PTB33 [*bph2*, *Bph3*, *BPH32*], five South Asian landraces that have been widely used in rice breeding programs aimed at producing planthopper-resistant varieties [36,39]. We included Triveni and Utri Rajapan from India and Indonesia respectively, as landraces with noted tolerance to planthoppers and the viruses they transmit [40,41,42]. In summary, we used three susceptible varieties (T65, TN1, IR22), 11 resistant varieties (Asominori, IR40, IR65482-7, IR62, IR65482-4, IR46, TKM6, Mudgo, ASD7, Rathu Heenati and PTB33) and two tolerant varieties (Triveni and Utri Rajapan).

### 2.3. Planthopper Colonies

We collected brown planthopper from rice fields near Santa Cruz in Laguna Province, Philippines. Over 500 adults were collected and caged with TN1 rice plants (≥30 DAS). Emerging F1 nymphs were separated from the adults to synchronize development and were multiplied through five generations on TN1 to build up numbers and to eliminate possible vectors of rice ragged stunt virus (RRSV) and rice grassy stunt virus (RGSV). The planthopper population from southern Laguna Province has been widely studied and is known to include a high proportion of individuals with virulence against *Bph1*, *bph2*, *bph5*, *bph7*, *bph8*, *BPH18*, *BPH25*, and *BPH26* [43]. All females used in our experiments were brachypterous. 

### 2.4. Bulk Test—SSST_7.8n_

For the SSST_7.8n_, we used seedboxes of 130 × 100 × 10 cm (L × W × H) filled with paddy soil to just below the rim of the box. Thirty seeds of each variety were sown in rows from one edge to the other along the width of the box and at a distance of 5 cm between adjacent rows. Seeds were sown to finger impressions in the soil (approx. 1 cm deep, 1 per hole) and covered with loose soil. Varieties, including TN1, were randomly assigned to rows. The susceptible check, TN1, was also sown in a central strip with eight varieties at either side, and again in a further two rows at either end of the seedbox. The seedlings were allowed to develop for 7 days, after which the plants were thinned to 20 per strip. At 7 days after sowing, the seedbox was infested with planthopper nymphs sprinkled over the plants at a density of 3040 per seedbox (8 nymphs per seedling). During the tests, a mesh cage of 140 × 120 × 100 cm (L × W × H) was fitted neatly over each seedbox. A second tray, with varieties placed in the same relative positions (i.e., duplicating the random assignment of lines) was set up at the same time but was not infested (i.e., non-infested control). This second tray was also covered with a mesh cage. Infested and non-infested seedboxes were replicated 6 times and were all infested at the same time in the greenhouse. The experiment was set up as a block design, with infested and corresponding non-infested control seedboxes representing blocks. Seedboxes were examined daily, noting damage to TN1 using the SES. When the TN1 rows in each seedbox had wilted due to planthopper feeding (i.e., SES rating = 9), the seedbox and associated control seedbox were destructively sampled. Rows were each assigned an SES rating. The numbers of living and dead plants per row were then counted and the plants were cut at soil level and placed in paper bags (each rice line from infested and control seedboxes was contained in a separate bag). The plants were dried at 60 °C in a forced draught oven for 1 week and were weighed.

### 2.5. Bulk Tests—MSST_7.2n_ and MSST_20.4n_

For the MSST_7.2n_, seedlings were sown in seedboxes (described above) and tended as described for the SSST_7.8n_ above. The seedlings were thinned to 20 plants per line before infestation. Seedlings were infested at a density of 2 × ≤2nd instar nymphs per plant (760 per seedbox) as described above. For the MSST_20.4n_, 20 seedlings were sown to seedboxes as described above and tended for 20 days. The seedlings were thinned to 10 plants per line at 15 DAS. The lower number of plants per row in this version of the MSST was to allow plants to grow larger as the plants were infested at 20 DAS (compared to 7 days for the SSST_7.8n_ and MSST_7.2n_). Seedlings were infested at a density of 4 × ≤2nd instar nymphs per plant (760 per seedbox) as described above. For both tests, the seedboxes were each covered with a screen cage (as described above). Non-infested, control seedboxes with the same configuration of test varieties were maintained to form experimental blocks. For both tests, the infested seedboxes and non-infested control seedboxes were replicated six times in the greenhouse (i.e., total = 24 seedboxes). Seedboxes were evaluated and destructively sampled as described in Section 2.4.

### 2.6. Days-to-Wilt Test (DTW_30.25n_)

Seeds of each variety were sown to soil impressions (1 cm deep) in pots (15 × 15 cm (H × D)) at a density of one seed per pot. The plants were allowed to grow and develop for 25 days after which the pots were covered with acetate cages of 112.5 × 11.5 cm (H × D) each with a mesh top and side window (23 × 14 cm). Acetate cages were used instead of mesh cages to reduce potential egg parasitism due to *Anagrus* spp. and *Oligocita* spp. (Hymenoptera). When the plants had reached 30 DAS, they were each infested with 25 × ≤2nd instar planthoppers. A set of control, non-infested plants was also maintained. These were sown and covered with acetate insect cages at the same time as the infested plants. The entire experiment was set up as a completely randomized design with 10 replicates set out on a greenhouse bench (i.e., 16 varieties × 2 treatments × 10 replicates = 320 pots). The nymphs on the infested plants were allowed to develop until plants showed initial yellowing (i.e., 32–57 days after infestation) at which time the planthoppers were collected by removing the mesh top of the cage and passing a vacuum sampler over the rice plant. Where plants showed no signs of wilting, the planthoppers were collected 60 days after infestation. The planthoppers collected from each plant were counted, dried at 60 °C for 1 week and weighed, and the developmental stages were recorded. We also noted the sex of adults and their wing forms. The time in days at which each plant wilted was noted (observations were made each morning before 9:00 h). After the insects had been removed, all plants were allowed to grow and develop until grain reached 80% maturity. Plants were then destructively sampled and plants were divided into roots, shoots and panicles. Plant parts were dried in a forced draught oven at 60 °C for 1 week and weighed. Panicles were separated into filled and unfilled grain, and the grain was counted and weighed. 

### 2.7. Survival_7.8n_ Test

We conducted nymph survival tests that corresponded to the SSST_7.8n_ by planting rice seed (one per pot) in individual pots of 5 × 6.5 cm (H × D). The pots were each covered by an acetate cage (45 × 5 cm, H × D) with a mesh top. At seven DAS, plants were infested with 8× ≤2nd instar planthoppers. A set of non-infested, caged plants, of the same age, were maintained as controls. The experiment was set up as a completely randomized design with six replicates (16 varieties × 2 treatments × 6 replicates = 192 pots). After the plants were infested, the nymphs were allowed to grow and develop for 15 days, after which the planthoppers were collected using a vacuum sampler and the plants were destructively harvested. The plants and planthoppers were separated, placed in paper bags, and dried at 60 °C for 1 week. Once dried, the plants were weighed. Planthoppers were counted and weighed and the development stages noted. 

### 2.8. Survival_20.4n_ and Survival_20.5f_ Tests

We conducted survival tests that corresponded to the MSST_20.4n_ test by planting rice seed from each variety in individual pots of 7 × 11 cm (H × D). At 15 DAS, the plants were each covered by an acetate cage of 61 × 10 cm (H × D) with a mesh top and window (6 × 5 cm). At 20 DAS, plants were infested either with 4 × ≤2nd instar planthoppers (2 seedlings per pot = 8 nymphs) or 5 newly emerged female planthoppers (1 seedling per pot = 5 adults). A set of non-infested, caged plants, of the same age, were maintained as controls. The two completely randomized experiments each had 6 replicates (Survival_20.4n_ test = 16 varieties × 2 treatments × 6 replicates = 192 pots; Survival_20.5f_ test = 16 varieties × 2 treatments × 6 replicates = 192 pots). Nymphs in the Survival_20.4n_ test were allowed to grow and develop for 15 days. The number of living adults in the Survival_20.5f_ test was monitored daily until all adults had died. At the end of the experiments, nymphs (for the Survival_20.4n_ test) were collected using a vacuum sampler and the plants (for both experiments) were destructively harvested. Plants and nymphs were placed in separate bags and dried at 60 °C for 1 week before being weighed. 

### 2.9. Build-up_7.2__ḟ_ and Build-up_20.4__ḟ_ Tests

For the Build-up_7.2__ḟ_ test, plants of each variety were sown in 7 × 11 cm (H × D) pots at a density of 2 plants per pot. The plants were allowed to grow and develop until 5 DAS, after which the pots were individually covered with acetate insect cages of 61 × 10 cm (H × D). At 7 DAS, the plants were infested with 2 recently emerged and mated, pre-oviposition females (4 per pot). For the Build-up_20.4__ḟ_ test, plants of each variety were sown to 15 × 15 cm (H × D) pots at a density of 1 plant per pot. The plants were allowed to grow and develop until 15 DAS, after which they were individually covered with an acetate insect cage 112.5 × 11.5 cm (H × D) with a mesh top and side window (6 × 5 cm). At 20 DAS, the plants were each infested with 4 newly emerged and mated females (pre-oviposition). For each experiment, sets of non-infested, caged plants of the same age were maintained as controls. The experiments were set up as completely randomized designs with 6 replicates (Build-up_7.2__ḟ_ test = 16 varieties × 2 treatments × 6 replicates = 192 pots; Build-up_20.4__ḟ_ test = 16 varieties × 2 treatments × 6 replicates = 192 pots). All plants were monitored for 15 days before destructive sampling. At sampling, the rice plants were cut at soil level, placed in separate paper bags and dried at 60 °C for 1 week. Planthoppers were collected, counted, and the developmental stages noted. Any adults were sexed and their wing condition noted. 

### 2.10. Honeydew_20.2__ḟ_ Test 

Twenty-day old seedlings of each variety in pots (1 per pot of 5 × 6.5 cm [H × D]) were infested with 2 recently emerged and mated female planthoppers (pre-oviposition) inside plastic feeding chambers (5 × 5 cm [H × D]). The chambers each had a hole at the base and top through which the rice plant was passed. A cotton plug was used to seal the top hole around the plant. The chambers confined the adults to the base of the test plants and were placed over Whatman no. 1 filter paper treated with Bromocresol green. The filter paper fitted neatly around the rice stem through a hole in the center of the paper and a slit to one side. After feeding for 24 h, the area of excreted honeydew spots on the treated filter papers was measured using Image-J software version 1.48 (National Institute of Health, Rockville, MD, USA). Honeydew spots were separated into blue-rimmed spots and white spots that represented phloem-derived and xylem-derived honeydew, respectively [44].

### 2.11. Oviposition_20.2__ḟ/m_ Test 

We compared oviposition using recently emerged, previously non-mated pairs (1 male, 1 female) of planthoppers on the test varieties. Plants were infested at 30 DAS in 15 × 15 cm (H × D) pots covered with acetate cages (112.5 × 11.5 cm (H × D). The planthoppers were allowed to feed and mate, and females allowed to oviposit on the plants for 9 days, at which time the adults were removed from the cages. The plants were assessed after a further 6 days. Any emerged nymphs were collected using a vacuum sampler and counted. The plants were then cut at soil level and carefully dissected under a stereo microscope (10× magnification) to locate egg batches. The number and location (relative to the leaf midrib) of egg batches were noted. Each egg batch was examined and the numbers of hatched and unhatched eggs were recorded. The unhatched eggs were examined to determine whether they contained fertile embryos. 

### 2.12. Recording Test Times and Costs 

The relative costs of phenotyping tests were divided into 3 main components. These were: (1) costs related to space usage; (2) costs related to materials used; and (3) costs related to the setup, maintenance and evaluation of tests. Because costs related to space will differ between institutes (depending on the price per unit area of greenhouse or bench space), we report space requirements in terms of m^2^ of greenhouse or laboratory space for each test (based on 1 replicate of 16 rice lines) over the duration of the test. Space requirements were estimated as the sum of the base areas of experimental arenas such as seedboxes, cages or pots, with a buffer distance of 5 cm between individual arenas. We report technical support costs in terms of the time required to complete tests (i.e., setup, scoring, counting and sorting of test materials during evaluation, weighing materials, and other procedures). We used test times because of differences in hourly salaries between institutes. Times were estimated by recording setup and processing times during the experiments using stop-watches. Individual researchers and technicians recorded times to complete tasks at a normal working pace. We also estimated the space, materials and times required to maintain planthopper colonies for routine screening and the costs related to screening during entomological support for rice breeding at IRRI over two successive years of operation. Further details of cost estimates are included with table and figure legends when reporting results.

### 2.13. Data Analyses

Bulk tests were evaluated using multivariate general linear models (GLM). To assess plant responses from these tests, we initially included replicates as a blocking factor (infested and non-infested), but this was removed where there was no significant block effect. The damage ratings and estimated plant weight losses for each variety from bulk tests were compared using Spearman’s correlations. Because the damage ratings and plant weight losses from the DTW test were largely bimodal, we did not apply Spearman’s correlations against corresponding bulk tests. Planthopper fitness parameters (survival × reproduction) and tolerance estimates from the performance tests were examined using univariate general linear models (GLM) with plant weight initially included as a covariate and removed when non-significant. Comparisons of plant weight losses per unit of insect weight in different bulk tests were examined using a two-way GLM. For all factorial analyses, data were transformed where required to meet homogeneity of variance requirements for parametric analyses and ranked where data could not meet requirements. Pairwise comparisons between varieties were conducted using Tukey tests. We also compared results from test lines against the susceptible check, TN1, using Duncan’s many-to-one comparisons. 

We used Spearman’s correlations to examine relations between damage ratings from bulk tests and weight loss or plant mortality from the same tests. Spearman’s correlations were also used to examine the relations between the results of bulk tests (damage ratings) and insect or plant response parameters as estimated from corresponding performance tests. Because plants were often severely damaged by the end of the performance tests, we assessed response parameters against damage ratings for all rice lines (N = 16) and again for those lines with damage ratings ≤7 (N = 11). We applied multiple linear regression with backward elimination to assess the best predictors of damage ratings and weight loss to plants from the performance tests. 

## 3. Results

### 3.1. Correlations between Results of SSST_7.8n_ and Survival_7.8n_ Test

The damage ratings from the SSST_7.8n_ were closely correlated with both the average weight loss of plants of each variety (Spearman’s *r_s_*_16_ = 0.916, *p* < 0.001) and the proportion of plants that had died (Spearman’s *r_s_*_16_ = 0.970, *p* < 0.001) (Table S1).

Results from the survival_7.8n_ test are presented in Table S2. The damage ratings and plant weight loss from the survival_7.8n_ test and SSST_7.8n_ were highly correlated (damage rating: Spearman’s *r_s_*_16_ = 0.893, *p* < 0.001; weight loss: Spearman’s *r_s_*_16_ = 0.687, *p* = 0.003); damage ratings were weakly correlated with planthopper biomass (Table 2). Planthopper survival appeared negatively correlated with SSST damage ratings; however, this was an artifact due to the declining quality of susceptible hosts during the test and the relation was not apparent among resistant and moderately resistant plants (i.e., df = 11: Table 2). There were no useful predictors from the survival_7.8n_ test for plant weight loss.

### 3.2. Correlations between MSST_7.2n_ and Build-up_7.2__ḟ_ Test

The damage rating from the MSST_7.2n_ was closely correlated with both the average weight loss of plants of each variety (Spearman’s *r_s_*_16_ = 0.886, *p* < 0.001) and the proportion of plants that had died (Spearman’s *r_s_*_16_ = 0.918, *p* < 0.001) (Table S3).

The damage rating from the MSST_7.2n_ was correlated with plant weight loss in the Build-up_7.2__ḟ_ test (Spearman’s *r_s_*_16_ = 0.687, *p* = 0.003); plant weight losses from both tests were also highly correlated (Spearman’s *r_s_*_16_ = 0.752, *p* < 0.001) (Table 2), but weight loss was generally not correlated with any of the parameters from the Build-up_7.2__ḟ_ test (i.e., number of planthoppers, planthopper biomass and planthopper development: Table S4). Damage ratings were weakly, negatively correlated with the proportion of early instars on the rice plants (Table 2). There were no significant predictors of plant weight loss from the Build-up_7.2__ḟ_ test.

### 3.3. Correlations between MSST_20.4n_ and Performance Tests with 20 DAS Plants 

The damage ratings from the MSST_20.4n_ were closely correlated with both the average weight loss of plants of each variety (Spearman’s *r_s_*_16_ = 0.866, *p* < 0.001) and the proportion of plants that had died (Spearman’s *r_s_*_16_ = 0.904, *p* < 0.001) (Table S5).

Results from performance tests corresponding to the MSST_20.4n_ are presented in Tables S6–S10. The damage ratings from the MSST_20.4n_ were weakly related to the weight and development of planthoppers from the Survival_20.4n_ test and closely related with the number and weight of planthoppers on non-wilted plants in the corresponding build-up test (Table 2). 

Damage ratings were weakly related to adult longevity on non-wilted plants but were highly correlated with the numbers of egg batches and total number of eggs produced by gravid females. Damage ratings were also related to the proportion of eggs that were infertile, and the total of unhatched eggs (Table 2). Damage ratings were negatively correlated with the average size of egg batches (Spearman’s *r_s_*_16_ = −0.501, *p* = 0.048) and positively correlated with the proportion of eggs that were laid along the midrib (Spearman’s *r_s_*_16_ = 0.532, *p* = 0.034) (Table S10). Damage ratings from the MSST_20.4n_ were not correlated with any of the parameters from the honeydew test (Table 2). 

The number of egg batches produced in the Oviposition_20.2__ḟ/m_ test was the best predictor of damage scores (F_1,15_ = 43.615, *p* < 0.001); meanwhile the development of nymphs to the adult stage in the Survival_20.4n_ test (F_1,15_ = 8.555, *p* = 0.011) and the number of planthoppers emerging in the Build-up_20.4__ḟ_ test (F_1,15_ = 7.603, *p* = 0.015) were the best predictors of plant weight loss in the tests. Parameters related to adult survival and honeydew production were not useful as predictors of plant weight loss or damage ratings. 

### 3.4. Correlations between DTW and Insect Responses

The damage ratings and time for plants to wilt during the DTW_30.25n_ test were highly correlated with the final numbers of planthoppers in the cages, the development of nymphs to adult, and the proportion of adults that were female (Table 3). Results were not significantly related to planthopper weight (Table 3). Development to adults, the proportion of adults that were female, and planthopper population size in the DTW_30.25n_ tests were also correlated with the MSST_20.4n_ damage ratings for the same varieties (Table 3). Higher proportions of females tended to occur on the more resistant plants (Table S11). The weight of planthoppers and the proportion of adults that were female were the best predictors of plant weight loss (F_2,15_ = 13.979, *p* < 0.001) and yield loss (F_2,15_ = 28.855, *p* < 0.001) as estimated during the DTW_30.25n_ tests (Table S12). 

### 3.5. Correlations between Results from Bulk Tests 

**Figure 3:**
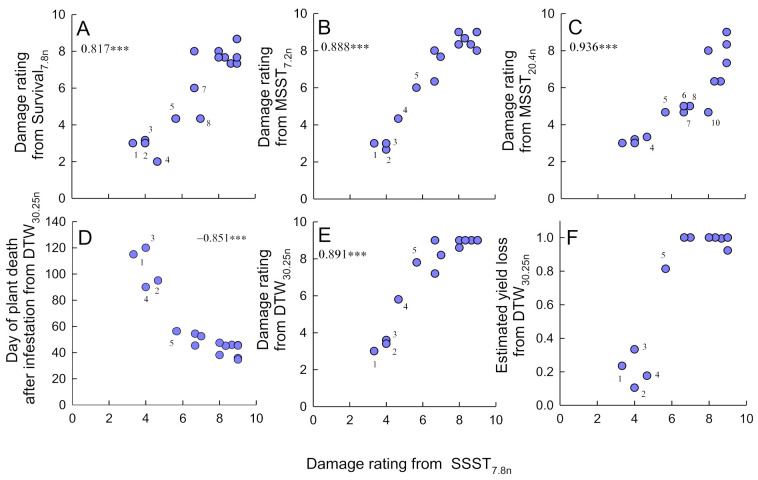
Bi-plots of SSST_7.8n_ damage ratings (x-axes) against damage ratings from (**A**) the Survival_7.8n_, (**B**) MSST_7.2n_, and (**C**) MSST_20.4n_ tests. Bi-plots of SSST_7.8n_ damage ratings against the evaluations from the DTW_30.25n_ test are presented as (**D**) the number of days for plants to senesce in the DTW_30.25n_ test, (**E**) the damage rating and (**F**) the proportional losses in yield during the DTW_30.25n_ test. Damage ratings are based on the standard evaluations systems (SES) as explained in Table 1. The results of Spearman’s correlations are indicated where *** *p* < 0.001. Symbol labels indicate rankings from the SSST_7.8n_ as 1 = Rathu Heenati, 2 = IR62, 3 = PTB33, 4 = IR65482-4, 5 = Triveni, 6 = IR46, 7 = Mudgo, 8 = IR65482-7, and 10 = Utri Rajapan.

Damage scores according to the SES were highly correlated across all bulk tests, the Survival_7.8n_ test, and the DTW_30.25n_ test (0.741 ≥ *r_s_*_16_ ≤ 0.936, *p* < 0.001) (Figure 3). The time for plants to wilt after infestation in the DTW_30.25n_ test was also highly correlated with damage ratings from all other tests (−0.924 ≥ *r_s_*_16_ ≤ -0.708, *p* < 0.001). Yield losses from the DTW test were also correlated with the damage ratings from all tests (0.646 ≥ *r_s_*_16_ ≤ 0.827, *p* < 0.01). Scores from the MSST_20.4n_ indicated IR46, Mudgo. IR65482-7, and Utri Rajapan as moderately resistant to the brown planthopper, but none of these lines had damage ratings below 6 in the SSST_7.8n_, MSST_7.2n_ or DTW_30.25n_ tests (Figure 3B–F).

**Figure 4:**
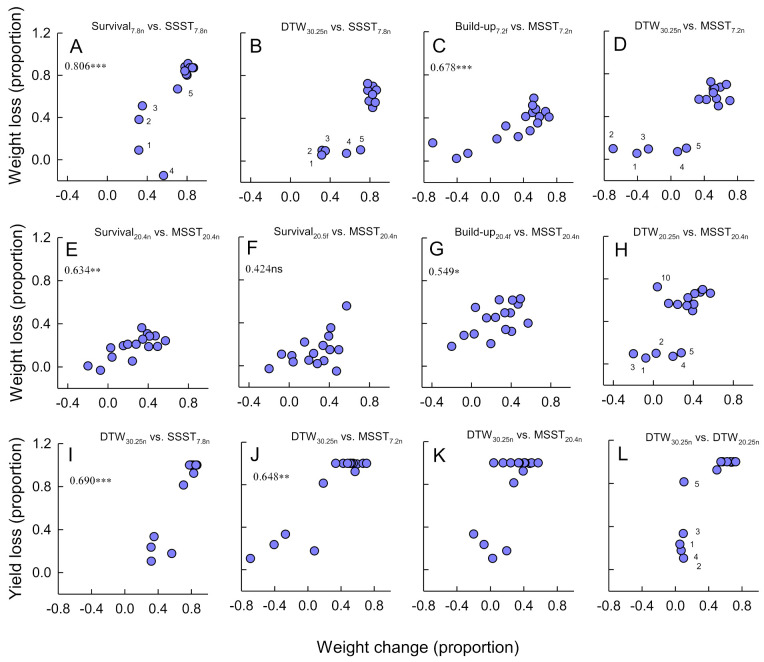
Bi-plots indicating (**A**–**K**) plant weight change during bulk tests with planthoppers (x-axes) and in corresponding performance tests (y-axes). A bi-plot of total plant weight loss and yield loss during the DTW tests is presented in **L**. Graphs include the results of (**A**) the Survival_7.8n_ and (**B**) DTW_30.25n_ plotted against results from the SSST_7.8n_, results from (**C**) the Build-up_7.2f_ and (**D**) DTW_30.25n_ against results from the MSST_7.2n_, and results from (**E**) the Survival_20.4n_, (**F**) Survival_20.5f_, (**G**) Build-up_20.4f_ and (**H**) DTW_30.25n_ tests against results from the MSST_20.4n_ test. Estimated losses to yields from the DTW_30.25n_ tests are plotted against plant weight loss from (**I**) the SSST_7.8n_, (**J**) the MSST_7.2n_, (**K**) the MSST_20.4n_ and (**L**) the DTW_30.25n_ tests. The results of Spearman’s correlations are indicated in (**A**,**C**,**E**,**G**,**I**–**L**) where *** *p* < 0.001, ** *p* < 0.01, * *p* < 0.05, and ns = *p* > 0.05. Note that negative values indicate overcompensation. Symbol labels indicate rankings from the SSST_7.8n_ as 1 = Rathu Heenati, 2 = IR62, 3 = PTB33, 4 = IR65482-4, 5 = Triveni and 10 = Utri Rajapan.

Weight loss from infested rice lines was often correlated across tests (Figure 4). Unlike damage ratings, records of weight loss could be used to identify lines that were highly tolerant of insect damage, or that overcompensated for insect feeding. Overcompensation in the growth of 20+ DAS seedlings was apparent in the MSST_7.2n_ and MSST_20.4n_ for Rathu Heenati, IR62 and PTB33, with relatively low weight loss in IR65482-4 and Triveni (Figure 4C–H). However, overcompensation was not apparent in any of the related performance tests (Figure 4C–H). Furthermore, there was no indication of overcompensation in plant weight or in yields during the DTW_30.25n_ tests (Figure 4B,D,H,I–L). Plant weight loss and loss in yield in the DTW_30.25n_ tests clearly separated rice lines into three groups as (1) lines with high weight loss, (2) lines (Rathu Heenati, IR62, PTB33, and IR65482-4) with low loss of plant weight and yields, and (3) Triveni, a line with relatively low plant weight loss, but a high loss of yield (Figure 4L).

**Figure 5:**
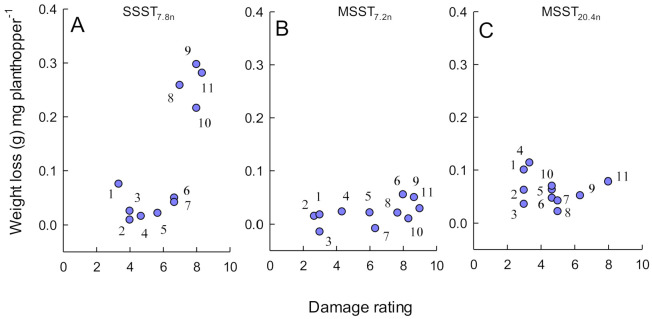
Bi-plots of plant weight loss per unit weight of planthopper estimated from (**A**) Survival_7.8n_ tests, (**B**) Build-up_7.2n_ tests and (**C**) Build-up_20.4n_ tests against damage ratings from equivalent bulk tests (A = SSST_7.8n_, B = MSST_7.2n_, C = MSST_20.4n_). Numbers are rice lines as follows: 1 = Rathu Heenati, 2 = IR62, 3 = PTB33, 4 = IR65482-4, 5 = Triveni, 6 = IR46, 7 = Mudgo, 8 = IR65482-7, 9 = IR40, 10 = Utri Rajapan, 11 = TKM6. All other lines were severely damaged by planthoppers before the end of the experiments and per-unit weight losses could not be calculated.

Weight loss to seedlings per unit weight of planthopper varied according to phenotyping test (F_2,150_ = 4.722, *p* = 0.010) (Figure 5). Varieties IR40, IR65482-7, Utri Rajapan, and TKM6 tended to have relatively high damage per unit weight gain of planthoppers compared to the remaining varieties, but only in the Survival_7.8n_ tests (interaction term: F_18,150_ = 1.866, *p* = 0.021), Across tests, weight losses per unit planthopper were similar (F_9,150_ = 1.724, *p* = 0.088). A correlation (*r_s_*_11_ = 0.737, *p* < 0.010) between per unit damage and SSST_7.8n_ results indicates that the damage rating for the test is highly affected by a lack of plant tolerance at early seedling stages (Figure 5A). Weight loss per unit weight of planthopper generally declined in the Build-up_7.2__ḟ_ and Build-up_20.4__ḟ_ tests (Figure 5B,C) indicating a greater tolerance to planthopper damage in older seedlings.

### 3.6. Phenotyping Costs

**Figure 6:**
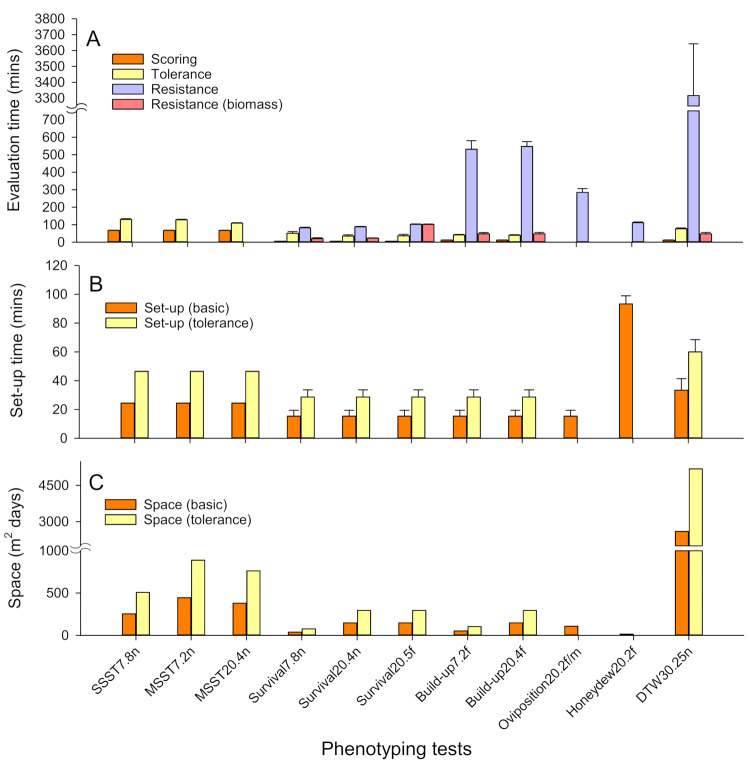
Relative time and space requirements for 11 phenotyping tests as implemented in the present study. (**A**) The time taken to evaluate phenotyping tests is presented for tests that were scored using the SES (i.e., damage ratings), for tests that included an evaluation of plant weight loss (as an indicator of tolerance), and for detailed evaluation of insect response variables (resistance). Time costs where counting and sorting could be replaced by drying and weighing of insects are also indicated (=resistance [biomass]). The time to set up bioassays is indicated in (**B**) with related space requirements in (**C**). Evaluation times were recorded during evaluation of the experiments in this study. Setup times for resistance tests were recorded during the present study with times to set up bulk testing estimated from normal operations at the IRRI entomology unit (2009–2015). All space and time requirements are for a single replicate of 16 test lines. Standard errors are indicated (N = 6 or 10).

The TN1 plants wilted more quickly in the SSST_7.8n_ (17.67 ± 2.06 days) test compared to the MSST_7.2n_ (37.67 ± 4.33 days) and MSST_20.4n_ tests (31.33 ± 1.23 days) (F_2,15_ = 12.776, *p* = 0.001). The type of test conducted affected the rating of damage using the SES (F_5,34_ = 176.801, *p* < 0.001). Survival tests were evaluated in ca 7 min, build-up and DTW tests in ca 14 min and bulk tests in ca 24 min (Figure 6, Table S13). Detailed, bionomic responses by planthoppers to rice lines took longer to evaluate (F_7,44_ = 44.940, *p* < 0.001) for the DTW_30.25n_ test than for the remaining tests (Survival tests, Build-up tests, Oviposition and Honeydew test) (Figure 6, Table S13). The times to evaluate planthopper responses from the herbivore-performance tests could be reduced by evaluating only the biomass of insects at the end of experiments (F_1,68_ = 86.527, *p* < 0.001) (Figure 6A). Weighting insects took less time for the Survival tests (22.3 ± 1.86 min) than for the DTW_30.25n_ (30.00 ± 1.29 min) and Build-up tests (42.9 ± 6.37 min) (Figure 6A). Weighing infested and control plants to assess plant weight losses was most rapid for the Survival, Build-up and DTW tests (57.85 ± 5.67 min), and took the longest for the SSST_7.8n_ and MSST_7.2n_ (123.80 ± 3.42 min). Because of fewer plants in the seedboxes, the MSST_20.4n_ plants could be weighed more quickly (83.33 ± 2.51 min) compared to the other bulk tests (F_8,49_ = 44.479, *p* < 0.001) (Table S13). 

Additional costs related to the tests included setup materials that ranged from $8 to $60 per 16 lines and setup times that ranged from 15 min for tests using potted rice plants to 93 min for honeydew tests (Figure 6B, Table S13). The maintenance of planthopper colonies is also a major time commitment during phenotyping. We estimated that the maintenance of three colonies, producing 40,000 nymphs every 32–35 days (sufficient for SSSTs with 4000 rice lines) requires 2987.06 ± 145.88 min (ca 50 h) of work for a technician each month. Recording of times from routine screening of rice lines at IRRI indicated that set up and equipment costs per line are substantially reduced as the numbers of lines to be tested increase (Figure S1). Space requirements were greatest for the DTW test and bulk tests and doubled whenever weight change responses (including tolerance) to insect infestations were estimated (Figure 6, Table S13). 

## 4. Discussion

The SSST is the most prevalent test used during the phenotyping of rice for resistance to planthoppers and leafhoppers. We estimate that over 75% of papers related to the breeding and genetics of rice resistance apply this test (Horgan, unpublished data). Its prevalence may be due to the relatively low cost of the SSST, particularly where very large numbers of rice lines are evaluated, as indicated in Figure 1 and Figure S1 based on routine screening at IRRI over two years. For example, based on a full cost recovery exercise at IRRI during 2010, we estimated that the cost of high-throughput screening (>500 lines per run) against the brown planthopper or green leafhopper using the SSST_7.8n_ was about $1 test^−1^ line^−1^ (Horgan and Almazan: unpublished report). However, several studies have identified shortcomings associated with the SSST [18,19,20,21,24,28]. These include a number of studies that indicate that the SSST will eliminate plants from breeding programs based on the susceptibility of seedlings under relatively high densities of planthopper nymphs, thereby overlooking ontogenetic changes in resistance and tolerance and possible low damage to older seedlings under lower and more realistic planthopper densities. For this reason, Velusamy et al. (1986) [20] suggested that bulk tests could be conducted using older plants and lower densities of planthoppers that would better indicate “field resistance” among lines. “Field resistance” refers to relative levels of accumulated damage to rice plants during sustained, low rates of herbivore feeding. In effect, either tolerance to insect damage (i.e., the ability of the rice plant to compensate for herbivore feeding) or true resistance (i.e., the ability of a plant to defend against herbivory) could underlie any observed “field resistance” [27,28,45].

### 4.1. Applying Adequate Tests

In our study, we found that results from two versions of the MSST were highly correlated with those of the SSST; the relative rankings of the least damaged varieties were maintained across all three bulk tests, but there was an improvement during the MSST_20.4n_ in the scores of many varieties that appeared susceptible in the other two tests. Meanwhile in our DTW_30.25n_ test, the four highest-ranking varieties (most “resistant”) from the bulk tests, also sustained the lowest damage from planthoppers through relatively late plant growth stages. In terms of separating varieties, the DTW_30,25n_ test showed the clearest distinction between useful, resistant or tolerant varieties and highly damaged susceptible varieties, both when evaluated based on the time at which the plants had wilted and when using the SES damage ratings. These results indicate that, depending on the age at which plants are evaluated and on the intensity of infestation, tolerant rice plants with relatively low resistance (such as Triveni [40]) are prone to elimination from breeding programs, with the SSST being least likely to identify tolerant lines because tolerance generally increases as plants age and gain size [27,45]. Because resistance is a relatively rare trait among rice plants [29,30], and because planthoppers and other herbivores occur in rice fields together with their natural enemies [9], then tests such as the MSST, which evaluates plants under moderate densities of herbivores, might be most suitable for screening studies aimed at low pesticide systems. We agree with Velusamy et al. (1986) [20] and recommend that, where possible, some version of the MSST should be applied during routine screening to better maintain tolerant lines in breeding programs. 

The application of SES damage ratings during research into the nature of rice-herbivore interactions (i.e., studies other than screening) is difficult to justify because the damage ratings cannot distinguish between antibiosis, antixenosis and/or tolerance. Bionomic tests that include choice tests and herbivore or plant performance tests are therefore applied for more detailed analyses of rice-herbivore interactions, including research into resistance mechanisms [28,29]. Bionomic tests will indicate the likely targets of resistance mechanisms. For example, in our oviposition tests, we clearly identified high egg mortality in the *japonica* lines Asominori and T65 (Table S10), two lines that were highly susceptible to planthopper nymphs and adults. The use of such detailed phenotyping tests is particularly beneficial for QTL analyses [46,47,48,49] and other research aimed at gene discovery [50,51] and where possible should substitute bulk tests. 

Plant tolerance to damage is a useful trait for farmers and can be highly advantageous for production systems that optimize ecologically-based pest management [7]. Tolerance is often non-specific and tolerant plants can often withstand attacks from a range of insects and diseases; furthermore, there is no selection pressure for planthoppers to adapt to tolerant varieties as occurs with resistant varieties [26,41,42]. A number of recent studies have indicated the benefits of assessing tolerance in rice to planthoppers and thereby distinguishing resistance from tolerance in rice plants [27,28,45]. Our results indicate that many plants will express both tolerance and resistance, sometimes during different plant growth stages. Because tolerance can be enhanced by resource availability (e.g., light, water, soil nutrients) and is related to plant growth rates, then combining resistance and tolerance in rice can be highly beneficial [7,27,52,53]. Therefore, we recommend that further attention to issues of plant tolerance be incorporated into breeding programs, particularly during phases where the numbers of rice lines are below about 100 (see Figure 1). However, to identify such potentially tolerant varieties in large breeding programs, an adequate bulk test, such as the MSST_20.4n_ is initially required. The assessment of tolerance in performance tests does imply increased phenotyping costs because plant materials and test arenas need to be doubled; however, plant weight loss is relatively easy to quantify and tolerance can therefore be quickly evaluated. 

### 4.2. Factors Determining Damage Ratings from Bulk Tests

High levels of damage to rice plants can be due to a high susceptibility or a low tolerance to herbivore attack. Bulk tests cannot distinguish these two factors. Indeed, our results suggest that bulk tests may actually exaggerate levels of plant tolerance. For example, our results based on plant weights indicated the least damaged plants in the MSSTs as overcompensating for planthopper damage. However, in performance tests—including the DTW test, there was no evidence of overcompensation in these same plants (Figure 4). One reason for these differences between tests is that severe damage to some of the plants in the bulk tests likely reduced competition for space between rice lines thereby allowing the most resistant plants to gain greater biomass in infested seedboxes compared to non-infested, control seedboxes. This indicates that the evaluation of relative tolerance is best conducted using herbivore performance tests with one variety per experimental unit. Furthermore, herbivore pressures need to be carefully quantified to get the best estimates of tolerance and plant roots must be uninhibited by pot size [52,53,54,55,56]. Herbivore feeding pressures cannot be quantified for different lines in bulk tests because planthoppers move between plants. In a study by Horgan et al. (2018) [27], resistance was a prerequisite for resource-induced tolerance. Therefore, rice plants that score high for resistance might generally express high tolerance also; indeed, evidence from our herbivore performance tests suggests that Rathu Heenati, IR62 and PTB33 are more tolerant of herbivore damage than were the remaining rice lines and this contributed to their low damage scores in the bulk tests. The plants that we included in this study to represent tolerant varieties rarely appeared more tolerant than these three highly resistant varieties. There was some indication that Triveni and IR65482-4 were tolerant of high numbers of planthoppers, but contrary to expectations, Utri Rajapan appeared to have a low tolerance. The appearance of tolerance also varied between tests. For example, IR65482-4 appeared highly tolerant to insect densities in bionomic evaluations associated with the DTW test, but not in other tests. To better assess tolerance, we recommend short-term exposures to planthoppers with evaluations conducted before any apparent yellowing of the plants, followed by assessments of weight changes to both the planthoppers and the plants. Planthopper-infested plants that appear healthy will undergo physiological changes that are not visible to the human eye [19]. The depletion of host quality due to extended periods of insect feeding can also increase intraspecific competition between planthoppers that distorts estimates of tolerance.

### 4.3. Novel Information from Herbivore Performance Tests

The SSST is based on challenging young rice seedlings with planthopper nymphs. Because the test is completed in 7 to 10 days, it does not incorporate other aspects of the planthopper life history. For example, there is no impact of adults on the plants and no measure of oviposition preferences among varieties. The MSSTs were designed to overcome these shortcomings [20]. However, our analysis of planthopper populations from Survival tests showed that few F1 individuals occurred in our MSSTs and that the resulting damage to plants was due to the original planthopper generation. FI planthoppers did affect the plants in the DTW test. Therefore, although the protocols for the MSST have been described in detail [20], some modifications to the tests may be required according to test conditions in different institutes to ensure the presence of F1 populations before the tests are completed. Among the rapid tests that we conducted, the DTW test most closely met the targets originally set out for the MSSTs. The results from the DTW in terms of the numbers and biomass of planthoppers at the end of the test, suggest that resistance in Rathu Heenati, IR62 and PTB33 affected nymphs and adults in a similar manner. This was further indicated by the Survival_20.5__ḟ_ test. The Ovipostion_20.2__ḟ/m_ test also indicated relatively low egg-laying on the same resistant plants. However, a recent, detailed study of oviposition in planthoppers indicates that much of the egg load of females is derived from the host on which the female develops, with subsequent egg-laying cycles determined by the food acquired during oviposition [57]. For example, in our oviposition test, the females were reared on TN1 before transfer to test lines and were therefore capable of laying large numbers of eggs during an initial oviposition cycle, even on the resistant varieties. The relatively lower numbers of eggs on the resistant varieties compared to susceptible varieties at the end of the test were due to the poor quality of the exposed host (the test variety), low adult survivorship, and few eggs laid during a second or third oviposition cycle on the test variety. Where F1 individuals develop on a resistant host, egg-laying should be considerably lower than was observed in our oviposition tests. Therefore, unless evaluated in a choice test, oviposition does appear to be generally related to antibiosis effects on nymphs. 

Poor quality of damaged, susceptible plants by the end of bionomic experiments often reduced planthopper fitness such that planthoppers displayed similar responses to susceptible and resistant plants by the end of the tests. For example, we found that the numbers of infertile eggs and unhatched eggs in our tests were positively correlated with damage according to the SES. This is likely due to the poor quality of damaged plants that affects egg survival. Nevertheless, the high egg mortality associated with *japonica* lines observed in the oviposition test does indicate how bulk tests can overlook some aspects of resistance. Egg mortality has been well-studied in rice, particularly against the white-backed planthopper [48,49]. Interestingly, the ovicidal response in *japonica* rice was first noted from field observations [58] and was not detected from bulk tests. We allowed plants in our herbivore performance tests to become severely damaged because we wished to relate the tests to the results of bulk tests; however, for best results, even moderate damage to host plants should be avoided during herbivore performance tests. This can be achieved by using older plants, or higher numbers of plants in pots, and by infesting at low planthopper densities. Herbivore-performance tests of shorter duration will also improve test results. 

Our honeydew tests indicated that planthoppers on TKM6 produced only small amounts of honeydew and displayed a relatively high consumption of xylem that is associated with feeding on a resistant host (Table S9); however, TKM6 appeared as susceptible as TN1 in all other tests. This may be because antibiosis is normally determined by both antifeeding and anti-assimilation mechanisms, but that planthoppers could otherwise compensate for poor host quality on TKM6, perhaps through high assimilation of food [59] and possibly aided by endosymbionts [44]. Although honeydew tests are commonly applied during phenotyping and are often regarded as providing quality information related to antibiosis [44], our results found little correlation between any of the parameters from honeydew tests and the results of the corresponding MSST_20.4n_. Among the parameters that were best correlated with damage ratings was planthopper weight (weight is also correlated with planthopper development and oviposition). We found that survival (without observations of adult emergence) is not a good indicator of resistance and that development stages are costly to record (Figure 6, Table S13)—particularly for DTW and Build-up tests. In contrast, recording and comparing planthopper biomass from plants that are not yet severely damaged is a relatively cheap assessment of resistance (Table S13). As more resistant varieties are examined using detailed phenotyping tests, we expect that a greater knowledge of the range of different host-herbivore interactions may be gained. 

### 4.4. The Relative Costs of Tests

Our assessment of the relative costs of different phenotyping tests indicates that bulk tests and the DTW test require the greatest amount of greenhouse space, the honeydew test and DTW test take longer to set up, and herbivore-performance tests generally require considerable time for detailed evaluations. Any assessment of plant tolerance will necessarily double space requirements and setup times, and adds to the time required to evaluate results. Our analysis of costs was based on replicates of 16 rice lines. However, the screening of large numbers of lines using bulk tests considerably reduces the per-line costs of screening (Figure S1), but not the costs of herbivore-performance tests. For example, where greater than 200 lines are assessed during routine screening with the SSST, preparation and disposal times are 4× lower, scoring times 2× lower, and tray space and soil amounts are reduced by 4× and 2× per line, respectively (Figure S1). These low costs and relatively simple protocols ensure that bulk tests are the most cost-effective methods to screen large numbers of rice lines. Despite the shortcomings of the tests as discussed above, bulk tests are nevertheless highly robust (Figure 3) and were successful in identifying useful plant materials to reduce planthopper and leafhopper damage in the field [36,60]. Nevertheless, our analysis indicates that bulk testing can be improved by switching from SSSTs to MSSTs with only a marginal increase in space-related costs—because the MSSTs take relatively longer to complete—but without increases in the costs of setting up the tests, and with reductions in the times taken to evaluate lines (Table S13). 

The DTW test gives clear results; however, the test is the most expensive of the rapid-evaluation tests. It requires a relatively large amount of space because older plants require larger pots, and the test can continue beyond 60 DAS. We do not recommend the DTW test for high-throughput screening, but it is highly useful in segregating resistant and susceptible plants where line numbers are small (e.g., <100 lines). The DTW test is also among the more rapidly evaluated tests when based on time to wilting or on the SES—damage rating. Evaluating insect performance across numerous accessions or lines is tedious and time-consuming compared with the bulk tests where plant impacts alone are assessed. Furthermore, some of these tests take a long time to evaluate (i.e., Build-up, Oviposition and Honeydew tests). However, by focusing only on biomass, the relative costs of evaluating tests such as Survival and Build-up tests can be substantially reduced. Therefore, we further recommend that donor varieties and pre-release lines (<100 lines) could be assessed for tolerance and for the nature of insect responses using Build-up tests with both insects and plants evaluated based on relative changes in biomass. Such changes would actually reduce space requirements, and reduce setup and evaluation costs compared to bulk tests, but do require some specialized equipment such as drying ovens and precision balances.

## 5. Conclusions

A suitable MSST that allows the development of F1 planthopper generations and thereby exposes test lines to ovipositing females, developing eggs and to feeding on late-stage rice plants is recommended over the use of the SSST during rice phenotyping with ≥100 lines. The SSST is highly affected by the lack of herbivory tolerance in young rice plants. Herbivore performance tests such as oviposition and population build-up tests are recommended to better assess antibiosis-based resistance, but such detailed tests can be costly compared to bulk tests. However, the costs associated with some of these performance tests can be reduced by evaluating changes to herbivore biomass only. Furthermore, by adding a non-infested control, performance tests can be used to assess rice plant tolerance. The DTW test was the most successful of the rapidly-evaluated tests for distinguishing susceptible and resistant/tolerant rice varieties and is recommended for phenotyping experiments with <100 lines. In particular, we recommend the DTW test for research leading to resistance gene discovery and for marker-assisted selection. 

## Figures and Tables

**Table 1:** Details of tests conducted in this study including the location (table number) of detailed results for each test in the supplementary information.

Test	Plant Age (Days)	No. of Seedlings Per Pot/Seedbox	Planthopper Density Plant^−1^	Insect Stage	Test Duration	Controls	Parameters Recorded ^1^	Results in Supplementary Tables
SSST_7.8n_	7	20 × 16 lines + 3 extra lines of TN1 = 380	8	≤2nd instar	When TN1 had died	Non-infested seedbox	Final plant dry weights (per row), number of living plants, SES damage rating	S1
MSST_7.2n_	7	20 × 16 lines + 3 extra lines of TN1 = 380	2	≤2nd instar	When TN1 had died	Non-infested seedbox	Final plant dry weights (per row), number of living plants, SES damage rating	S3
MSST_20.4n_	20	10 × 16 lines + 3 extra lines of TN1 = 190	4	≤2nd instar	When TN1 had died	Non-infested seedbox	Final plant dry weights (per row), number of living plants, SES damage rating	S5
DTW_30.25n_	30	1	25	≤2nd instar	60 days or when plants were about to die	Non-infested plants in pots	Days-to-wilt, SES damage ratings, Number of planthoppers per pot, planthopper development stages, adult sex, dry weight of planthoppers, final plant dry weights, weight of filled and unfilled grain,	S11, S12
Performance tests								
Survival_7.8n_	7	1	8	≤2nd instar	15 days	Non-infested plants in pots	Final plant dry weights, SES damage rating, remaining number of planthoppers, planthopper development stages, sex of adults, planthopper dry weight	S2
Survival_20.4n_	20	2	4	≤2nd instar	15 days	Non-infested plants in pots	Final plant dry weights, number of planthoppers remaining, planthopper development stages, sex of adults, adult wing forms, planthopper dry weight	S6
Survival_20.5f_	20	1	5	Newly-emerged virgin females	When all females had died	Non-infested plants in pots	Final plant dry weights, time to 50% adult dead, time to 100% adult death	S8
Build-up_7.2ḟ_	7	5	2	Gravid females	15 days	Non-infested plants in pots	Final plant weights, number of planthoppers, planthopper development stages, dry weight of planthoppers	S4
Build-up_20.4ḟ_	20	1	4	Gravid females	15 days	Non-infested plants in pots	Final plant weights, number of planthoppers, planthopper development stages, dry weight of planthoppers	S7
Honeydew_20.2ḟ_	20	1	2	Gravid females	24 h	None	Area of acidic and basic honeydew spots, number of acidic and basic honeydew spots	S9
Oviposition_20.2ḟ/m_	20	1	2	1 adult male, 1 adult female	15 days	None	Location of egg batches, number of egg batches, number of eggs per batch, number of emerged nymphs, number of unhatched fertile and infertile eggs	S10

^1.^The standard evaluation system (SES) rates planthopper damage in bulk tests as follows: ‘0’ = ‘no damage’, ‘1’ = ‘slight damage’ (high resistance [HR]), ‘3’ = ‘1st and 2nd leaves of most plants damaged’ (resistance [R]), ‘5’ = ‘pronounced yellowing and stunting with ≤25% of plants stunted or dead’ (moderate resistance [MR]), ‘7’ = ‘≥50% of plants dead (moderately susceptible’ [MS]), and ‘9’ = ‘all plants dead’ (susceptible [S]). Corresponding ratings for individual plants in performance tests are ‘0’ = ‘no visible damage’, ‘1’ = ‘partial yellowing of first leaf’, ‘3’ = ‘1st and 2nd leaves partially yellow’, ‘5’ = ‘pronounced yellowing or some stunting’, ‘7’ = ‘wilted or severely stunted’, ‘9’ = ‘plant dead’.

**Table 2:** Spearman’s coefficients (*r*_*s*16_ and *r*_*s*11_) for correlations between damage ratings from bulk tests and herbivore-response parameters estimated from corresponding performance tests.

Bulk Tests	Corresponding Performance Tests ^1^	Herbivore-Performance Test Parameters ^2^			
SSST_7.8n_	Survival_7.8n_	Proportion surviving	Proportion at adult stage	Proportion of adults that were female	Weight of planthoppers
	[16]	−0.720 **	−0.086ns	0.263ns ^3^	−0.564 *
	[11]	0.218ns	0.388ns	−0.137ns	−0.085 *
MSST_7.2n_	Build-up_7.2n_	Proportion at 1st instar	Number of planthoppers	-	Weight of planthoppers
	[16]	−0.485ns	0.010ns		0.215ns
	[11]	−0.572 *	0.193ns		0.404ns
MSST_20.4n_	Survival_20.4n_	Proportion surviving	Proportion at adult stage	Proportion of adults that were female	Weight of planthoppers
	[16]	0.280ns	0.564 *	−0.183ns ^3^	0.510 *
	[11]	0.206ns	0.773 **	0.122ns	0.712 *
MSST_20.4n_	Build-up_20.4f_	Proportion at 1st instar	Number of planthoppers	-	Weight of planthoppers
	[16]	−0.125ns	0.443ns		0.399ns
	[11]	−0.381ns	0.940 ***		0.884 ***
MSST_20.4n_	Survival_20.5f_	Time to 50% mortality	Time to 100% mortality	*-*	*-*
	[16]	0.176ns	0.458ns		
	[11]	0.282ns	0.684 *		
MSST_20.4n_	Oviposition_20.2f/m_	Number of batches	Number of eggs	Infertile eggs	Unhatched eggs
	[16]	0.885 ***	0.817 ***	0.740 ***	0.591 *
	[11]	0.856 ***	0.609 *	0.557ns	0.671 *
MSST_20.4n_	Honeydew_20.2f_	Area of phloem	Area of xylem	Total honeydew area	Xylem as a proportion of total
	[16]	0.047ns	−0.194ns	0.016ns	−0.147ns
	[11]	0.242ns	0.098ns	0.121ns	−0.098ns

^1.^Numbers in square brackets are degrees of freedom for Spearman’s correlations. Correlations were tested for all 16 varieties and for 11 varieties with damage ratings of ≤7 during tests. ^2.^Herbivore-performance test parameters are indicated as sub-headings. Means for each parameter and variety are presented in Tables S1–S10. Numbers are Spearman’s correlation coefficients; *** = *p* < 0.001, ** = *p* < 0.01, * = *p* < 0.05, ns = *p* > 0.05. ^3.^N = 15 because no adults emerged on T65.

**Table 3:** Spearman’s coefficients (*r_s_*_16_) for correlations between damage ratings and time for plants to wilt in the DTW_30.25n_ test and herbivore- and plant-response parameters estimated from the same test. Correlations with MSST_20.4n_ damage ratings are also shown.

MSST and DTW Test and Corresponding Evaluation Method ^1^	Planthopper Responses ^2^				Plant Responses ^2^		
	Proportion at Adult Stage	Proportion of Adults That Were Female	Total Number of Planthoppers	Pooled Weight of Planthoppers	Plant Weight Loss as a Proportion	Plant Yield Loss as a Proportion	Actual Weight Loss of Plants
DTW_30.25n_—damage rating	−0.722 ***	−0.800 ***	0.597 **	0.289ns	0.850 ***	0.928 ***	0.769 ***
DTW_30.25n_—days	0.714 ***	0.770 ***	−0.546 *	−0.074ns	−0.760 ***	−0.757 ***	−0.658 **
MSST_20.4n_—damage rating	−0.713 ***	−0.715 ***	0.532 *	−0.115ns	0.733 ***	0.730 ***	0.704 ***

^1.^ Rapid evaluation of the DTW_30.25n_ test can be conducted using damage ratings (i.e., SES scores) or by noting the day after infestation at which plants had died. ^2.^ Numbers are Spearman’s correlation coefficients (N = 16); *** = *p* < 0.001, ** = *p* < 0.01, * = *p* < 0.05, ns = *p* > 0.05.

## Data Availability

Data in support of results from this study are presented as supplementary information.

## Supplementary Materials

The following are available online at https://www.mdpi.com/article/10.3390/insects12100847/s1, Figure S1: Time to process rice lines during routine SSST with brown planthopper at IRRI., Table S1: Results from the SSST_7.8n_ test with 16 rice lines, Table S2: Results from nymph Survival_7.8n_ test with 16 varieties, Table S3: Results from MSST_7.2n_ with 16 rice lines, Table S4: Results from Build-up_7.2__ḟ_ test corresponding to MSST_7.2n_, Table S5: Results from MSST_20.4n_ with 16 rice lines, Table S6: Results from nymph Survival_20.4n_ test with 16 varieties, Table S7: Results from Build-up_20.4__ḟ_ test corresponding to MSST_20.4n_, Table S8: Adult longevity on 16 rice lines according to the Survival_20.5__ḟ_ test, Table S9: Honeydew production on 16 rice lines based on the Honeydew_20.2__ḟ_ test, Table S10: Results from Oviposition_20.2__ḟ/m_ test on 16 rice lines, Table S11: Results from Days to Wilt (DTW_30.25n_) test indicating responses by planthoppers to 16 rice lines, Table S12: Results from Days to Wilt (DTW_30.25n_) test indicating responses by 16 rice lines to infestation by planthoppers, Table S13: Estimated costs and time for processing materials during 11 phenotyping tests.

## References

[1] Otuka A. (2013). Migration of rice planthoppers and their vectored re-emerging and novel rice viruses in East Asia. Front. Microbiol..

[2] Zhou G., Xu D., Xu D., Zhang M. (2013). Southern rice black-streaked dwarf virus: A white-backed planthopper-transmitted fijivirus threatening rice production in Asia. Front. Microbiol..

[3] Thresh J. (1989). Insect-borne viruses of rice and the Green Revolution. Int. J. Pest. Manag..

[4] Kumar S., Singh H., Yadav A., Kumar A., Shanker R. (2019). Seasonal abundance of brown plant hopper, *Nilaparvata lugens* (Stal) in basmati rice and correlation of abiotic factors under Meerut region. J. Entomol. Zool. Stud..

[5] Bottrell D.G., Schoenly K.G. (2012). Resurrecting the ghost of green revolutions past: The brown planthopper as a recurring threat to high-yielding rice production in tropical Asia. J. Asia-Pac. Entomol..

[6] Wu J., Ge L., Liu F., Song Q., Stanley D. (2020). Pesticide-induced planthopper population resurgence in rice cropping systems. Annu. Rev. Entomol..

[7] Horgan F.G. (2018). Integrating gene deployment and crop management for improved rice resistance to Asian planthoppers. Crop. Prot..

[8] Hu C., Guo J., Fu X., Huang Y., Gao X., Wu K. (2018). Seasonal migration pattern of *Nilaparvata lugens* (Hemiptera: Delphacidae) over the Bohai Sea in Northern China. J. Econ. Entomol..

[9] Horgan F.G., Crisol Martínez E., Stuart A.M., Bernal C.C., de Cima Martín E., Almazan M.L.P., Ramal A.F. (2019). Effects of vegetation strips, fertilizer levels and varietal resistance on the integrated management of arthropod biodiversity in a tropical rice ecosystem. Insects.

[10] Sattler C., Schrader J., Flor R.J., Keo M., Chhun S., Choun S., Hadi B.A.R., Settele J. (2021). Reducing pesticides and increasing crop diversification offer ecological and economic benefits for farmers—A case study in Cambodian rice fields. Insects.

[11] Fujita D., Kohli A., Horgan F.G. (2013). Rice resistance to planthoppers and leafhoppers. Crit. Rev. Plant Sci..

[12] Sani Haliru B., Rafii M.Y., Mazlan N., Ramlee S.I., Muhammad I.I., Silas Akos I., Halidu J., Swaray S., Rini Bashir Y. (2020). Recent strategies for detection and improvement of brown planthopper resistance genes in rice: A review. Plants.

[13] Satturu V., Vattikuti J.L., Durga Sai J., Kumar A., Singh R.K., Srinivas P.M., Zaw H., Jubay M.L., Satish L., Rathore A. (2020). Multiple genome wide association mapping models identify quantitative trait nucleotides for brown planthopper (*Nilaparvata lugens*) resistance in MAGIC *indica* population of rice. Vaccines.

[14] Horgan F.G., Almazan M.L.P., Vu Q., Ramal A.F., Bernal C.C., Yasui H., Fujita D. (2019). Unanticipated benefits and potential ecological costs associated with pyramiding leafhopper resistance loci in rice. Crop. Prot..

[15] He L., Zou L., Huang Q., Sheng X., Wu W., Hu J. (2020). Development of InDel markers of *Bph3* and pyramiding of four brown planthopper resistance genes into an elite rice variety. Mol. Breed..

[16] Han Y., Wu C., Yang L., Zhang D., Xiao Y. (2018). Resistance to *Nilaparvata lugens* in rice lines introgressed with the resistance genes *Bph14* and *Bph15* and related resistance types. PLoS ONE.

[17] Wang H., Ye S., Mou T. (2016). Molecular breeding of rice restorer lines and hybrids for brown planthopper (BPH) resistance using the *Bph14* and *Bph15* genes. Rice.

[18] Soundararajan R., Jeyaprakash P. (2019). Rapid screening of rice genotypes for resistance to brown planthopper, *Nilaparvata lugens* (Stål). Electron. J. Plant. Breed..

[19] Horgan F.G., Jauregui A., Peñalver Cruz A., Crisol Martínez E., Bernal C.C. (2020). Changes in reflectance of rice seedlings during planthopper feeding as detected by digital camera: Potential applications for high-throughput phenotyping. PLoS ONE.

[20] Velusamy R., Heinrichs E.A., Medrano F.G. (1986). Greenhouse techniques to identify field resistance to the brown planthopper, *Nilaparvata lugens* (Stål) (Homoptera: Delphacidae), in rice cultivars. Crop. Prot..

[21] Chen Y., Li S., Li R. (2010). Comparative study on evaluation methods for resistance to *Nilaparvata lugens* Stål in rice. J. Anhui Agric. Sci..

[22] Horgan F.G., Heong K.L., Hardy B. (2009). Mechanisms of resistance: A major gap in understanding planthopper-rice interactions. Planthoppers: New Threats to the Sustainability of Intensive Rice Production Systems in Asia.

[23] Sarao P.S., Bentur J.S. (2018). Quantification of antibiosis levels in nine different rice genotypes against *Nilaparvata lugens* (Homoptera: Delphacidae). Int. J. Trop. Insect Sci..

[24] Sogawa K., Qian Q., Zeng D.-L.Z., Hu J., Zeng L.-J. (2005). Differential expression of whitebacked planthopper resistance in the *japonica/indica* doubled haploid rice population under field evaluation and seedbox screening test. Rice Sci..

[25] Smith C.M., Clement S.L. (2012). Molecular bases of plant resistance to arthropods. Annu. Rev. Entomol..

[26] Peterson R.K.D., Varella A.C., Higley L.G. (2017). Tolerance: The forgotten child of plant resistance. PeerJ.

[27] Horgan F.G., Peñalver Cruz A., Bernal C.C., Ramal A.F., Almazan M.L.P., Wilby A. (2018). Resistance and tolerance to the brown planthopper, *Nilaparvata lugens* (Stål), in rice infested at different growth stages across a gradient of nitrogen applications. Field Crops Res..

[28] Qiu Y., Guo J., Jing S., Tang M., Zhu L., He G. (2011). Identification of antibiosis and tolerance in rice varieties carrying brown planthopper resistance genes. Entomol. Exp. Appl..

[29] Heinrichs E.A. (1985). Genetic Evaluation for Insect Resistance in Rice.

[30] Heinrichs E.A. (1994). Biology and Management of Rice Insects.

[31] Liu G., Zhang Y., Gui L. (1999). On the screening methods for resistance to rice planthoppers (Homoptera: Delphacidae) in some Chinese rice varieties. Acta Agric. Zhejiangensis.

[32] Tao L. (1995). Studies on screening techniqes of resistance of rice varieties to brown planthopper (BPH), *Nilaparvata lugens* Stål. Acta Agric. Zhejiangensis.

[33] Pathak M., Cheng C., Fortuno M. (1969). Resistance to *Nephotettix impicticeps* and *Nilaparvata lugens* in varieties of rice. Nature.

[34] Huang Z., He G., Shu L., Li X., Zhang Q. (2001). Identification and mapping of two brown planthopper resistance genes in rice. Theor. Appl. Genet..

[35] Soundararajan R.P., Kadirvel P., Gunathilagaraj K., Maheswaran M. (2004). Mapping of quantitative trait loci associated with resistance to brown planthopper in rice by means of a doubled haploid population. Crops Sci..

[36] Horgan F.G., Srinivasan T.S., Bentur J.S., Kumar R., Bhanu K.V., Sarao P.S., Chien H.V., Almazan M.L.P., Bernal C.C., Ramal A.F. (2017). Geographic and research center origins of rice resistance to Asian planthoppers and leafhoppers: Implications for rice breeding and gene deployment. Agronomy.

[37] Horgan F.G., Srinivasan T.S., Naik B.S., Ramal A.F., Bernal C.C., Almazan M.L.P. (2016). Effects of nitrogen on egg-laying inhibition and ovicidal response in planthopper-resistant rice varieties. Crop. Prot..

[38] Khush G., Virk P. (2005). IR varieties and their impact. Rice Res. Inst., Los Baños, Philippines. IR Varieties and their Impact.

[39] Kim S.-M., Sohn J.-K. (2005). Identification of a rice gene (*Bph 1*) conferring resistance to brown planthopper (*Nilaparvata lugens* Stål) using STS markers. Mol. Cells.

[40] Ho D.T., Heinrichs E.A., Medrano F. (1982). Tolerance of the rice variety Triveni to the brown planthopper, *Nilaparvata lugens*. Environ. Entomol..

[41] Panda N., Heinrichs E.A. (1983). Levels of tolerance and antibiosis in rice varieties having moderate resistance to the brown planthopper, *Nilaparvata lugens* (Stål) (Hemiptera: Delphacidae). Environ. Entomol..

[42] Panda N., Heinrichs E.A., Hibino H. (1984). Resistance of the rice variety Utri Rajapan to ragged stunt and tungro viruses. Crop. Prot..

[43] Horgan F.G., Ramal A.F., Bentur J.S., Kumar R., Bhanu K.V., Sarao P.S., Iswanto E.H., Chien H.V., Phyu M.H., Bernal C.C. (2015). Virulence of brown planthopper (*Nilaparvata lugens*) populations from South and South East Asia against resistant rice varieties. Crop. Prot..

[44] Ferrater J.B., Naredo A.I., Almazan M.L.P., de Jong P.W., Dicke M., Horgan F.G. (2015). Varied responses by yeast-like symbionts during virulence adaptation in a monophagous phloem-feeding insect. Arthropod-Plant. Interact..

[45] Horgan F.G., Crisol-Martínez E., Almazan M.L.P., Romena A., Ramal A.F., Ferrater J.B., Bernal C.C. (2016). Susceptibility and tolerance in hybrid and pure-line rice varieties to herbivore attack: Biomass partitioning and resource-based compensation in response to damage. Ann. Appl. Biol..

[46] Alam S.N., Cohen M.B. (1998). Detection and analysis of QTLs for resistance to the brown planthopper, *Nilaparvata lugens*, in a doubled-haploid rice population. Theor. Appl. Genet..

[47] Cohen M.B., Alam S.N., Medina E.B., Bernal C.C. (1997). Brown planthopper, *Nilaparvata lugens*, resistance in rice cultivar IR64: Mechanism and role in successful *N. lugens* management in Central Luzon, Philippines. Entomol. Exp. Appl..

[48] Yamasaki M., Tsunematsu H., Yoshimura A., Iwata N., Yasui H. (1999). Quantitative trait locus mapping of ovicidal response in rice (*Oryza sativa* L.) against whitebacked planthopper (*Sogatella furcifera* Horváth). Crops Sci..

[49] Yamasaki M., Yoshimura A., Yasui H. (2000). Mapping of quantitative trait loci of ovicidal response to brown planthopper (*Nilaparvata lugens* Stål) in rice (*Oryza sativa* L.). Breed. Sci..

[50] Tamura Y., Hattori M., Yoshioka H., Yoshioka M., Takahashi A., Wu J., Sentoku N., Yasui H. (2014). Map-based cloning and characterization of a brown planthopper resistance gene *BPH26* from *Oryza sativa* L. ssp indica cultivar ADR52. Sci. Rep..

[51] Naik S.B., Divya D., Sahu N., Sundaram R.M., Sarao P.S., Singh K., Lakshmi V.J., Bentur J.S. (2018). A new gene *Bph33(t)* conferring resistance to brown planthopper (BPH), *Nilaparvata lugens* (Stål) in rice line RP2068-18-3-5. Euphytica.

[52] Liu J.-L., Yu J.-F., Wu J.-C., Yin J.-L., Gu H.-N. (2008). Physiological responses to *Nilaparvata lugens* in susceptible and resistant rice varieties: Allocation of assimilates between shoots and roots. J. Econ. Entomol..

[53] Qui H., Wu J., Yang G., Dong B., Li D. (2004). Changes in the uptake function of the rice root to nitrogen, phosphorus and potassium under brown planthopper, *Nilaparvata lugens* (Stål) (Homoptera: Delphacidae) and pesticide stresses, and effect of pesticides on rice grain filling in field. Crop. Prot..

[54] Wise M.J., Abrahamson W.G. (2005). Beyond the compensatory continuum: Environmental resource levels and plant tolerance of herbivory. Oikos.

[55] Wise M.J., Abrahamson W.G. (2007). Effects of resource availability on tolerance of herbivory: A review and assessment of three opposing models. Am. Nat..

[56] Crisol E., Almazan M.L.P., Jones P.W., Horgan F.G. (2013). Planthopper-rice interactions: Unequal stresses on pure-line and hybrid rice under similar experimental conditions. Entomol. Exp. Appl..

[57] Horgan F.G., Arida A., Ardestani G., Almazan M.L.P. (2021). Elevated temperatures diminish the effects of a highly resistant rice variety on the brown planthopper. Sci. Rep..

[58] Suzuki Y., Sogawa K., Seino Y. (1996). Ovicidal reaction of rice plants against the whitebacked planthopper, *Sogatella furclfera* Horváth (Homoptera: Delphacidae). Appl. Entomol. Zool..

[59] Seo B.Y., Jung J.K., Choi B.-R., Park H.M., Lee B.H., Heong K.L., Hardy B. (2009). Resistance-breaking ability and feeding behavior of the brown planthopper, *Nilaparvata lugens*, recently collected in Korea. Planthoppers: New Threats to the Sustainability of Intensive Rice Production Systems in Asia.

[60] Widawsky D., Rozelle S., Jin S., Huang J. (1998). Pesticide productivity, host-plant resistance and productivity in China. Agric. Econ..

