# Supplementary material for: Efficacy and Cost-Effectiveness of Phenotyping for Rice Resistance and Tolerance to Planthoppers
Source: Insects. 2021 Sep 22;12(10):847. doi: 10.3390/insects12100847 (PMC8539731; doi:10.3390/insects12100847)
Supplement: Supplementary file 1 [file insects-12-00847-s001.zip › Insects 1351897 Supp Info.pdf]

Supplementary information

Efficacy and cost-effectiveness of phenotyping for rice resistance and tolerance to planthoppers

Finbarr G. Horgan [1,2,*], Enrique A. Mundaca [2], Reyeul Quintana [3], Alberto I. Naredo [3], Maria Liberty P. Almazan [3] and Carmencita C. Bernal [3]

1. EcoLaVerna Integral Restoration Ecology, Bridestown, Kildinan, Co. Cork, Ireland
2. Universidad Católica del Maule, Facultad de Ciencias Agrarias y Forestales, Escuela de Agronomía, Casilla 7-D, Curicó, Chile
3. International Rice Research Institute, DAPO Box 7777, Metro Manila, Philippines *

Correspondence: f.horgan@ecolaverna.org

Table S1: Results from the SSST$_{7.8n}$ test with 16 rice lines. The SSST$_{7.8n}$ is a bulk test that is rapidly evaluated using damage ratings.

Rice Line	Plant weight (mg)(control)[2]	Plant weight (mg)(infested)[2]	Average plant weight Loss (proportion)[1,2]	Plant death (proportion)[2]	Damage rating[2,3]
Rathu Heenati	3.41±0.76 ^{ab}	2.48±0.92	**0.32±0.16 ^{a}**	0.25±0.09 ^{ab}	**3.33±0.33 ^{a}**
IR62	2.82±0.46 ^{ab}	2.14±0.71	**0.32±0.19 ^{ab}**	0.28±0.13 ^{a}	**4.00±0.45 ^{ab}**
PTB33	4.33±1.08 ^{ab}	3.13±1.13	**0.35±0.16 ^{abc}**	0.22±0.16 ^{ab}	**4.00±1.00 ^{abc}**
IR65482-4	3.25±0.53 ^{ab}	1.41±0.44	0.56±0.09 ^{abcd}	0.28±0.12 ^{ab}	**4.67±0.61 ^{abc}**
Triveni	2.92±0.45 ^{ab}	0.75±0.22	0.71±0.08 ^{abcde}	0.61±0.13 ^{abc}	**5.67±0.42 ^{abc}**
IR46	2.81±0.51 ^{ab}	0.47±0.12	0.81±0.04 ^{bcde}	0.70±0.11 ^{bcd}	**6.67±0.80 ^{bcd}**
Mudgo	5.89±1.17 ^{b}	1.33±0.43	0.79±0.04 ^{bcde}	0.60±0.14 ^{abcd}	**6.67±0.95 ^{cde}**
IR65482-7	2.81±0.68 ^{ab}	0.46±0.07	0.80±0.03 ^{bcde}	0.68±0.11 ^{bcd}	**7.00±0.52 ^{cde}**
IR40	1.68±0.28 ^{a}	0.31±0.06	0.78±0.04 ^{bcde}	0.77±0.07 ^{cde}	8.00±0.45 ^{def}
Utri Rajapan	3.18±0.51 ^{ab}	0.67±0.15	0.78±0.02 ^{abcde}	0.83±0.07 ^{cde}	8.00±0.45 ^{def}
TKM6	2.83±0.71 ^{ab}	0.39±0.10	0.83±0.04 ^{cde}	0.68±0.15 ^{cde}	8.33±0.42 ^{def}
ASD7	5.12±0.91 ^{ab}	0.97±0.24	0.81±0.02 ^{cde}	0.94±0.04 ^{cde}	8.67±0.33 ^{ef}
Asominori	2.07±0.32 ^{a}	0.30±0.06	0.83±0.03 ^{de}	0.97±0.02 ^{de}	9.00±0.00 ^{f}
IR22	3.03±0.73 ^{ab}	0.38±0.08	0.83±0.04 ^{e}	0.97±0.02 ^{cde}	9.00±0.00 ^{f}
T65	1.94±0.31 ^{a}	0.19±0.01	0.87±0.03 ^{e}	1.00±0.00 ^{e}	9.00±0.00 ^{f}
TN1	3.32±0.94 ^{ab}	0.38±0.07	0.85±0.02 ^{e}	1.00±0.00 ^{e}	9.00±0.00 ^{f}
F-value[4]	2.477***		5.854***	12.813***	19.106***

1: Average plant weight loss is calculated as (weight of control plants – weight of infested plants)//weight of control plants

2: Numbers are means ± SEM; lowercase letters indicate homogenous groups based on Tukey tests ($P > 0.05$); results indicated in bold font are significantly less affected by planthoppers than the susceptible check TN1 based on Duncan's many-to-one tests ($P > 0.05$)

3: Damage ratings are based on the standard evaluations systems (SES) for bulk tests as outlined in Table 1

4: DF = 15,80; *** = $P \leq 0.005$ (N = 6)

Table S2: Results from nymph Survival$_{7.8n}$ test with 16 varieties. The Survival$_{7.8n}$ test is a performance test that corresponds to the SSST7.8n in terms of plant age and insect densities

Rice line	Plant weight (control)(g)[2]	Plant weight (control)(g)[2]	Plant weight loss (proportion)[1,2]	Damage rating[2,3]	Survival (proportion)[2]	Development (proportion)[2]				Female (proportion)[2]	Pooled weight of planthoppers (mg)[2,3]
						3rd instar	4th instar	5th instar	Adult		
Rathu Heenati	0.11±0.02	0.10±0.04	**0.09±0.24 [a]**	**3.00±1.26 [ab]**	0.71±0.11 [abc]	0.19±0.16	0.10±0.05	0.10±0.07	0.67±0.16	0.42±0.14	1.86±0.66
IR62	0.09±0.01	0.06±0.01	**0.38±0.11 [a]**	**3.17±0.91 [ab]**	0.46±0.18 [abc]	0.00±0.00	0.08±0.05	0.25±0.25	0.68±0.24	0.45±0.23	2.78±1.08
PTB33	0.13±0.03	0.05±0.01	**0.51±0.08 [a]**	**3.00±0.00 [ab]**	0.69±0.10 [c]	0.11±0.08	0.11±0.08	0.00±0.00	0.88±0.06	0.41±0.06	2.97±1.22
IR65482-4	0.09±0.02	0.07±0.00	**-0.35±0.66 [a]**	**2.00±0.45 [a]**	0.69±0.12 [abc]	0.02±0.02	0.08±0.05	0.00±0.00	0.90±0.08	0.47±0.14	2.62±0.90
Triveni	0.10±0.02	0.02±0.00	0.67±0.11 [ab]	**4.33±0.42 [abc]**	0.88±0.08 [c]	0.00±0.00	0.00±0.00	0.03±0.03	0.97±0.03	0.49±0.08	3.31±0.96
IR46	0.12±0.02	0.00±0.00	0.91±0.02 [b]	8.00±0.68 [cd]	0.46±0.11 [abc]	0.13±0.13	0.00±0.00	0.05±0.05	0.90±0.07	0.51±0.08	2.53±1.27
Mudgo	0.14±0.03	0.02±0.00	0.81±0.05 [ab]	6.00±1.00 [bcd]	0.69±0.12 [bc]	0.06±0.06	0.00±0.00	0.00±0.00	0.93±0.07	0.44±0.07	2.94±1.41
IR65482-7	0.11±0.01	0.01±0.00	0.80±0.04 [ab]	**4.33±0.99 [abc]**	0.52±0.11 [abc]	0.00±0.00	0.00±0.00	0.17±0.17	0.83±0.17	0.53±0.06	3.34±1.17
IR40	0.09±0.01	0.01±0.00	0.88±0.03 [ab]	8.00±1.00 [cd]	0.27±0.09 [abc]	0.00±0.00	0.00±0.00	0.00±0.00	1.00±0.00	0.37±0.14	2.33±1.00
Utri Rajapan	0.12±0.03	0.01±0.00	0.84±0.04 [ab]	7.67±0.67 [cd]	0.63±0.14 [abc]	0.08±0.08	0.00±0.00	0.00±0.00	0.92±0.08	0.73±0.10	2.21±0.86
TKM6	0.10±0.01	0.01±0.00	0.87±0.04 [ab]	7.67±0.84 [cd]	0.27±0.11 [abc]	0.00±0.00	0.25±0.25	0.00±0.00	0.75±0.25	0.30±0.07	1.85±0.88
ASD7	0.13±0.03	0.02±0.00	0.87±0.05 [ab]	7.33±0.80 [cd]	0.75±0.09 [bc]	0.00±0.00	0.00±0.00	0.00±0.00	1.00±0.00	0.43±0.05	2.84±1.13
Asominori	0.07±0.02	0.01±0.00	0.87±0.06 [ab]	7.33±1.09 [cd]	0.33±0.11 [abc]	0.00±0.00	0.25±0.17	0.00±0.00	0.75±0.17	0.44±0.14	1.53±1.08
IR22	0.09±0.01	0.01±0.00	0.87±0.07 [ab]	8.67±0.33 [d]	0.15±0.06 [ab]	0.00±0.00	0.00±0.00	0.25±0.25	0.75±0.25	0.72±0.15	0.73±0.75
T65	0.07±0.01	0.01±0.00	0.87±0.08 [ab]	8.67±0.33 [a]	0.02±0.02 [a]	1.00±0.00	0.00±0.00	0.00±0.00	0.00±0.00	na	0.14±0.00
TN1	0.09±0.02	0.01±0.00	0.87±0.09 [ab]	7.67±0.67 [cd]	0.25±0.14 [abc]	0.00±0.00	0.00±0.00	0.33±0.33	0.83±0.17	0.75±0.13	2.46±1.13
F-line[4]	1.273ns		1.756*	8.802***	4.502***				1.099ns	1.604ns	1.548ns
F-covariate[4]			10.447***		3.894*						

1: Plant weight loss is calculated as (weight of control plants – weight of infested plants)/weight of control plants

2: Numbers are means ± SEM; lowercase letters indicate homogenous groups based on Tukey tests ($P > 0.05$); results indicated in bold font are significantly less affected by planthoppers than the susceptible check TN1 based on Duncan's many-to-one tests ($P > 0.05$)

3: Damage ratings are based on the standard evaluations system (SES) for individual plants as outlined in Table 1

4: DF = 15,80 or 15,79 where the covariate plant weight had a significant effect; *** = $P \leq 0.005$, * = $P \leq 0.05$, ns = $P \geq 0.05$ (N = 6)

Table S3: Results from $MSST_{7.2n}$ with 16 rice lines. The $MSST_{7.2n}$ is a bulk test that is rapidly evaluated using damage ratings.

Rice line	Plant weight (mg)(control)[2]	Plant weight (mg)(infested)[2]	Average plant weight loss (proportion)[1,2]	Plant death (proportion)[2]	Damage rating[2,3]
Rathu Heenati	10.75±2.24 [abc]	14.36±1.66	**-0.41±0.13** [ab]	**0.00±0.00** [a]	**3.00±0.00** [a]
IR62	7.45±1.28 [ab]	12.24±2.79	**-0.69±0.23** [a]	**0.01±0.03** [a]	**2.67±0.33** [a]
PTB33	12.72±2.22 [abc]	16.00±2.65	**-0.27±0.13** [a]	**0.00±0.00** [a]	**3.00±0.00** [a]
IR65482-4-136-2-2	8.94±1.22 [abc]	8.49±1.55	0.08±0.08 [abc]	0.11±0.10 [a]	**4.33±0.67** [ab]
Triveni	8.28±1.84 [ab]	7.12±2.62	0.19±0.10 [abcd]	0.46±0.18 [ab]	6.00±1.13 [bc]
IR46	8.26±1.36 [ab]	3.53±0.68	0.55±0.08 [cdef]	0.72±0.14 [bc]	8.00±0.68 [cd]
Mudgo	20.32±2.18 [c]	13.27±2.61	0.34±0.08 [abcde]	0.51±0.10 [ab]	6.33±0.67 [bc]
IR65482-7-216-1-2-B	8.32±1.95 [ab]	4.33±0.74	0.43±0.08 [bcdef]	0.80±0.08 [bc]	7.67±0.67 [cd]
IR40	6.01±1.78 [a]	2.37±0.51	0.51±0.09 [cdef]	1.00±0.00 [d]	9.00±0.00 [d]
Utri Rajapan	12.16±2.23 [abc]	6.16±1.29	0.48±0.05 [cdef]	0.85±0.08 [bcd]	8.33±0.42 [cd]
TKM6	8.79±2.15 [ab]	3.33±0.74	0.51±0.11 [def]	0.92±0.05 [cd]	8.67±0.33 [d]
ASD7	17.76±1.92 [bc]	7.24±1.50	0.59±0.05 [ef]	0.92±0.07 [cd]	8.33±0.42 [cd]
Asominori	6.66±1.50 [a]	2.17±0.26	0.57±0.09 [ef]	0.96±0.03 [cd]	9.00±0.00 [d]
IR22	8.32±1.67 [ab]	2.23±0.28	0.67±0.07 [f]	0.99±0.01[d]	9.00±0.00 [d]
Taichung 65	5.93±1.42 [a]	2.82±1.52	0.52±0.12 [ef]	0.87±0.10 [cd]	8.00±0.68 [cd]
Taichung Native 1	11.99±1.74 [abc]	3.45±0.40	0.71±0.05 [f]	0.84±0.16 [cd]	9.00±0.00 [d]
F-value[4]	4.357***		11.738***	20.675***	20.199***

1: Average plant weight loss is calculated as (weight of control plants– weight of infested plants)/weight of control plants; a negative value indicates a gain in weight after exposure to insects
2: Numbers are means ± SEM; lowercase letters indicate homogenous groups based on Tukey tests ($P > 0.05$), results indicated in bold font are significantly less affected by planthoppers than the susceptible check TN1 based on Duncan's many-to-one tests ($P > 0.05$)
3: Damage ratings are based on the standard evaluations system (SES) for bulk tests as outlined in Table 1
4: DF = 15,80; *** = $P \leq 0.005$ (N = 6)

Table S4: Results from Build-up$_{7.2f}$ test. The Build-up$_{7.2f}$ test is a performance test that corresponds to the MSST$_{7.2n}$ bulk tests because the bulk test was designed to permit nymphs to develop to the adult stage.

Rice line	Plant weight (g)(control)[2]	Plant weight (g)(infested)[2]	Weight loss (proportion)[1,2]	Development (proportion at each instar)[2]				Number of planthoppers[2]	Weight of planthoppers[2] (mg)
				1st instar	2nd instar	3rd instar	4th instar		
Rathu Heenati	0.29±0.04	0.25+0.29	**0.02±0.23** ^{a}	**0.67±0.12** ^{b}	0.22±0.06	0.12±0.07	0.00±0.00	186.83±73.21	2.66±0.99
IR62	0.28±0.03	0.22+0.28	0.17±0.07 ^{ab}	0.49±0.09 ^{ab}	0.28±0.03	0.18±0.08	0.05±0.04	225.67±58.42	4.35±0.97
PTB33	0.32±0.05	0.28+0.32	**0.07±0.11** ^{ab}	0.34±0.04 ^{ab}	0.50±0.08	0.13±0.04	0.02±0.01	219.83±53.95	4.76±0.96
IR65482-4	0.32±0.03	0.24+0.31	0.20±0.08 ^{ab}	0.41±0.08 ^{ab}	0.32±0.05	0.25±0.11	0.01±0.01	205.00±42.70	4.65±1.18
Triveni	0.28±0.04	0.17+0.28	0.32±0.15 ^{ab}	0.40±0.07 ^{ab}	0.32±0.02	0.24±0.07	0.04±0.01	363.61±43.27	6.04±0.91
IR46	0.58±0.33	0.15+0.57	0.48±0.10 ^{ab}	0.36±0.03 ^{ab}	0.34±0.05	0.26±0.06	0.04±0.02	312.50±49.12	6.83±0.98
Mudgo	0.40±0.04	0.30+0.39	0.23±0.11 ^{ab}	0.38±0.07 ^{ab}	0.34±0.05	0.28±0.08	0.01±0.00	225.17±72.40	4.79±1.32
IR65482-7	0.28±0.03	0.15+0.27	0.41±0.11 ^{ab}	0.39±0.07 ^{ab}	0.34±0.06	0.24±0.03	0.03±0.01	308.00±52.65	6.90±0.79
IR40	0.25±0.03	0.12+0.24	0.45±0.07 ^{ab}	0.29±0.06 ^{a}	0.34±0.03	0.32±0.04	0.05±0.02	211.67±58.24	5.10±1.15
Utri Rajapan	0.29±0.02	0.20+0.28	0.28±0.13 ^{ab}	0.28±0.04 ^{a}	0.41±0.09	0.28±0.05	0.03±0.01	332.00±66.22	6.68±1.08
TKM6	0.33±0.02	0.15+0.32	0.52±0.05 ^{ab}	0.36±0.05 ^{ab}	0.36±0.06	0.24±0.03	0.05±0.01	202.33±41.58	4.37±0.81
ASD7	0.37±0.03	0.22+0.36	0.41±0.05 ^{ab}	0.31±.02 ^{a}	0.35±0.02	0.32±0.03	0.03±0.01	297.33±26.03	6.43±1.50
Asominori	0.28±0.03	0.17+0.28	0.35±0.07 ^{ab}	0.37±0.08 ^{ab}	0.35±0.05	0.26±0.05	0.02±0.01	108.83±30.60	3.26±0.62
IR22	0.27±0.03	0.14+0.26	0.46±0.10 ^{ab}	0.37±0.04 ^{ab}	0.28±0.05	0.31±0.04	0.04±0.02	262.33±62.40	5.27±0.64
T65	0.26±0.03	0.09+0.25	0.58±0.06 ^{b}	0.53±0.07 ^{ab}	0.28±0.03	0.18±0.04	0.02±0.01	202.00±34.81	3.32±0.49
TN1	0.24±0.03	0.13+0.23	0.41±0.13 ^{ab}	0.40±0.05 ^{ab}	0.37±0.02	0.22±0.05	0.01±0.01	265.50±57.71	5.24±1.09
F-line[3]	0.884ns		2.489***	1.902*	1.492ns	0.891ns	1.057ns	1.154ns	1.691ns
F-covariate[3]			11.022***						

1: Average plant weight loss is calculated as (weight of control plants – weight of infested plants)/weight of control plants; a negative value indicates a gain in weight after exposure to insects
2: Numbers are means ± SEM; lowercase letters indicate homogenous groups based on Tukey tests ($P > 0.05$); results indicated in bold font are significantly less affected by planthoppers than the susceptible check TN1 based on Duncan's many-to-one tests ($P > 0.05$)
3: DF = 15,80; *** = $P \leq 0.005$; * = $P \leq 0.05$; ns = $P > 0.05$ (N = 6)

Table S5: Results from $MSST_{20.4n}$ with 16 rice lines. The $MSST_{20.4n}$ is a bulk test that is rapidly evaluated using damage ratings.

Rice line	Plant weight (mg)(control)[2]	Plant weight (mg)(infested)[2]	Average plant weight loss (proportion)[1,2]	Plant death (proportion)[2]	Damage rating[2,3]
Rathu Heenati	13.42±1.29 ^{cdef}	14.38±2.61	**-0.08±0.15 ^{ab}**	0.02±0.02 ^{ab}	**3.00±0.00 ^{a}**
IR62	11.69±0.66 ^{bcde}	11.35±0.79	0.03±0.09 ^{ab}	0.00±0.00 ^{a}	**3.00±0.00 ^{a}**
PTB33	18.54±3.16 ^{efg}	20.49±2.40	**-0.20±0.17 ^{a}**	0.18±0.16 ^{ab}	**3.00±0.00 ^{a}**
IR65482-4	14.38±0.99 ^{def}	11.39±0.98	0.20±0.07 ^{abc}	0.03±0.02 ^{ab}	**3.33±0.33 ^{ab}**
Triveni	13.85±1.01 ^{cdef}	9.46±0.86	0.28±0.13 ^{abc}	0.09±0.07 ^{ab}	**4.67±0.33 ^{bc}**
IR46	12.30±1.24 ^{bcde}	9.59±1.00	0.15±0.14 ^{abc}	0.09±0.05 ^{ab}	**4.67±0.33 ^{bc}**
Mudgo	29.33±4.22 ^{g}	16.04±1.50	0.41±0.09 ^{bc}	0.31±0.06 ^{abcd}	**5.00±0.00 ^{c}**
IR65482-7	11.41±1.16 ^{bcde}	8.18±1.17	0.24±0.14 ^{abc}	0.32±0.14 ^{ab}	**5.00±0.52 ^{c}**
IR40	7.44±0.33 ^{abc}	4.21±0.53	0.41±0.09 ^{bc}	0.91±0.06 ^{e}	8.00±0.45 ^{de}
Utri Rajapan	14.63±1.66 ^{def}	12.86±1.32	0.04±0.14 ^{ab}	0.12±0.07 ^{ab}	**4.67±0.33 ^{bc}**
TKM6	10.58±0.57 ^{abcde}	6.84±0.88	0.35±0.10 ^{abc}	0.30±0.14 ^{abcd}	**6.33±0.67 ^{cd}**
ASD7	22.65±1.90 ^{fg}	12.28±1.61	0.47±0.05 ^{bc}	0.28±0.12 ^{abc}	**6.33±0.67 ^{cd}**
Asominori	6.84±0.33 ^{ab}	4.20±0.68	0.39±0.11 ^{abc}	0.81±0.08 ^{cde}	**7.33±0.61 ^{de}**
IR22	11.34±0.49 ^{bcde}	5.51±0.44	0.49±0.04 ^{bc}	0.93±0.04 ^{e}	8.33±0.42 ^{e}
T65	5.79±0.77 ^{a}	2.29±0.35	0.57±0.04 ^{c}	0.85±0.14 ^{e}	9.00±0.00 ^{e}
TN1	10.09±2.92 ^{abcd}	6.43±1.79	0.34±0.05 ^{abc}	0.87±0.13 ^{de}	9.00±0.00 ^{e}
F-rice line[4]	11.438***		5.286***	13.715***	40.446***
F-covariate[4]			5.551*		

1: Average plant weight loss is calculated as (weight of control plants – weight of infested plants)/weight of control plants; a negative value indicates a gain in weight after exposure to insects

2: Numbers are means ± SEM; lowercase letters indicate homogenous groups based on Tukey tests ($P > 0.05$), results indicated in bold font are significantly less affected by planthoppers than the susceptible check TN1 based on Duncan's many-to-one tests ($P > 0.05$)

3: Damage ratings are based on the standard evaluations system (SES) for bulk tests as outlined in Table 1

4: DF = 15,80; *** = $P \leq 0.005$ (N = 6)

Table S6: Results from nymph Survival$_{20.4n}$ test with 16 varieties. The Survival$_{20.4n}$ test is a performance test that corresponds to the MSST$_{20.4n}$.

Rice line	Plant weight (mg)(control)^{2}	Plant weight (mg)(infested)^{2}	Plant weight loss (proportion)	Survival (proportion)^{2}	Development (proportion at each life stage)				Female (proportion)	Male long-winged (proportion)	Pooled planthopper weight (mg)
					3rd instar	4th instar	5th instar	Adult			
Rathu Heenati	1.04±0.13 ^{ab}	0.99±0.05	**-0.03±0.14 ^{a}**	0.75±0.08	0.02±0.02	0.20±0.09	0.23±0.08	**0.55±0.16 ^{a}**	0.22±0.11	0.86±0.10	**2.45±0.89 ^{a}**
IR62	0.99±0.03 ^{ab}	0.81±0.08	0.18±0.08 ^{ab}	0.92±0.04	0.00±0.00	0.00±0.00	0.13±0.13	0.88±0.13 ^{b}	0.43±0.12	1.00±0.00	**3.30±0.18 ^{ab}**
PTB33	1.05±0.05 ^{ab}	1.04±0.07	**0.01±0.06 ^{ab}**	0.73±0.11	0.00±0.00	0.06±0.04	0.04±0.04	0.90±0.08 ^{b}	0.60±0.09	1.00±0.00	3.66±0.91 ^{ab}
IR65482-4	1.01±0.07 ^{ab}	0.77±0.06	0.21±0.08 ^{ab}	0.88±0.06	0.00±0.00	0.00±0.00	0.02±0.02	0.98±0.02 ^{b}	0.60±0.13	1.00±0.00	3.80±0.81 ^{ab}
Triveni	0.98±0.08 ^{ab}	0.76±0.03	0.21±0.05 ^{ab}	0.79±0.11	0.00±0.00	0.02±0.02	0.00±0.00	0.98±0.02 ^{b}	0.71±0.08	1.00±0.00	5.81±1.39 ^{bc}
IR46	0.93±0.06 ^{ab}	0.74±0.05	0.20±0.04 ^{ab}	0.81±0.06	0.00±0.00	0.00±0.00	0.00±0.00	1.00±0.00 ^{b}	0.67±0.04	0.69±0.16	6.37±0.70 ^{bc}
Mudgo	1.28±0.06 ^{b}	1.02±0.04	0.19±0.05 ^{ab}	0.92±0.04	0.00±0.00	0.00±0.00	0.00±0.00	1.00±0.00 ^{b}	0.57±0.08	1.00±0.00	4.83±0.90 ^{abc}
IR65482-7	0.93±0.05 ^{ab}	0.86±0.03	**0.05±0.05 ^{ab}**	0.81±0.14	0.00±0.00	0.00±0.00	0.00±0.00	1.00±0.00 ^{b}	0.66±0.09	0.79±0.09	5.43±1.42 ^{abc}
IR40	0.82±0.11 ^{a}	0.58±0.09	0.28±0.05 ^{ab}	0.71±0.11	0.00±0.00	0.00±0.00	0.00±0.00	1.00±0.00 ^{b}	0.47±0.12	0.77±0.12	5.14±1.11 ^{abc}
Utri Rajapan	1.10±0.09 ^{ab}	1.00±0.13	0.09±0.10 ^{ab}	0.88±0.06	0.00±0.00	0.00±0.00	0.00±0.00	1.00±0.00 ^{b}	0.67±0.12	0.78±0.15	7.19±1.40 ^{c}
TKM6	1.08±0.04 ^{ab}	0.79±0.06	0.26±0.07 ^{ab}	0.88±0.06	0.00±0.00	0.00±0.00	0.00±0.00	1.00±0.00 ^{b}	0.56±0.06	0.87±0.06	6.56±0.58^{bc}
ASD7	1.19±0.20 ^{ab}	0.86±0.15	0.29±0.04 ^{ab}	0.79±0.06	0.00±0.00	0.00±0.00	0.00±0.00	1.00±0.00 ^{b}	0.51±0.13	0.93±0.07	6.17±1.18 ^{bc}
Asominori	0.85±0.06 ^{ab}	0.57±0.02	0.31±0.05 ^{ab}	0.98±0.02	0.00±0.00	0.00±0.00	0.00±0.00	1.00±0.00 ^{b}	0.54±0.09	0.89±0.07	5.13±0.80 ^{abc}
IR22	0.91±0.12 ^{ab}	0.71±0.06	0.19±0.06 ^{ab}	0.85±0.07	0.00±0.00	0.00±0.00	0.02±0.02	0.98±0.02 ^{b}	0.43±0.07	0.97±0.03	5.48±1.00 ^{abc}
T65	0.82±0.06 ^{a}	0.60±0.06	0.24±0.08 ^{ab}	0.92±0.05	0.00±0.00	0.00±0.00	0.00±0.00	1.00±0.00 ^{b}	0.47±0.06	0.87±0.06	5.49±0.56 ^{abc}
TN1	1.09±0.06 ^{ab}	0.68±0.09	0.36±0.09 ^{b}	0.90±0.04	0.00±0.00	0.00±0.00	0.00±0.00	1.00±0.00 ^{b}	0.60±0.05	0.86±0.09	7.09±0.73 ^{c}
F-rice line	2.095*		2.937***	1.073ns	-	-	-	5.020***	1.318ns	1.469ns	2.155*
F-covariate			12.188***								6.116*

1: Plant weight loss is calculated as (weight of control plants – weight of infested plants)/weight of control plants

2: Numbers are means ± SEM; lowercase letters indicate homogenous groups based on Tukey tests (P > 0.05); results indicated in bold font are significantly less affected by planthoppers than the susceptible check TN1 based on Duncan's many-to-one tests (P > 0.05)

3: DF = 15,80 or 15,79 where the covariate plant weight had a significant effect; *** = P ≤ 0.005, * = P ≤ 0.05, ns = P ≥ 0.05; - = no test due to insufficient variability; (N = 6)

Table S7: Results from Build-up$_{20.4f}$ test. The Build-up$_{20.4f}$ is a performance test that corresponds to the MSST$_{20.4n}$.

Rice line	Plant weight (mg)(control)[2]	Plant weight (mg)(infested)[2]	Plant weight loss (proportion)	Development (proportion at each life stage)				Number of planthoppers	Pooled planthopper weight (mg)
				1st instar	2nd instar	3rd instar	4th instar		
Rathu	0.92±0.10 ab		0.29±0.15 abc	**0.68±0.16 b**	0.16±0.06	0.15±0.12	0.00±0.00	**92.50±42.30 ab**	**2.38±1.23 a**
Heenati		0.60±0.09							
IR62	0.93±0.09 ab	0.63±0.08	0.30±0.09 abc	0.51±0.06 ab	0.35±0.03	0.13±0.05	0.01±0.01	281.17±69.14 abc	5.14±1.10 ab
PTB33	0.87±0.09 ab	0.69±0.11	**0.18±0.13 a**	**0.62±0.13 b**	0.34±0.14	0.04±0.03	0.00±0.00	145.33±40.37 abc	**2.40±0.54 a**
IR65482-4	0.91±0.10 ab	0.68±0.09	0.21±0.13 ab	0.43±0.11 a	0.34±0.08	0.22±0.14	0.01±0.01	129.33±45.61 abc	3.47±1.06 ab
Triveni	0.77±0.05 ab	0.29±0.03	0.62±0.03 bc	0.52±0.07 ab	0.31±0.03	0.14±0.04	0.04±0.02	316.33±63.67 abc	7.17±1.45 ab
IR46	0.86±0.14 ab	0.44±0.08	0.45±0.12 abc	0.33±0.07 a	0.30±0.06	0.33±0.12	0.04±0.02	298.50±49.39 abc	8.81±1.28 ab
Mudgo	0.95±0.06 ab	0.64±0.06	0.32±0.05 abc	0.37±0.06 a	0.42±0.06	0.19±0.04	0.03±0.01	331.00±40.69 abc	11.57±1.61 ab
IR65482-7	0.93±0.16 ab	0.40±0.05	0.45±0.13 abc	0.39±0.07 a	0.41±0.05	0.18±0.06	0.02±0.01	364.83±68.69 bc	11.56±3.50 ab
IR40	0.60±0.08 a	0.17±0.03	0.62±0.15 bc	0.46±0.09 a	0.31±0.03	0.20±0.06	0.03±0.02	409.33±58.59 c	10.30±2.68 ab
Utri Rajapan	1.09±0.15 b	0.47±0.08	0.55±0.08 abc	**0.67±0.09 b**	0.25±0.06	0.07±0.03	0.01±0.01	311.50±61.38 abc	6.40±1.35 ab
TKM6	0.68±0.14 a	0.32±0.04	0.34±0.26 abc	0.53±0.10 ab	0.37±0.09	0.09±0.03	0.01±0.01	398.51±75.31 c	10.65±2.08 ab
ASD7	1.11±0.11 b	0.42±0.03	0.58±0.08 abc	0.47±0.11 a	0.33±0.05	0.18±0.07	0.01±0.01	394.50±53.04 c	10.10±2.49 ab
Asominori	0.75±0.09 a	0.37±0.09	0.50±0.11abc	**0.73±0.10 b**	0.21±0.07	0.05±0.03	0.01±0.01	136.17±23.62 abc	**2.67±0.69 a**
IR22	0.99±0.10 ab	0.35±0.06	0.63±0.07 c	0.43±0.09 a	0.30±0.03	0.24±0.08	0.03±0.01	410.50±82.30 c	12.76±4.13 b
T65	0.63±0.08 a	0.35±0.06	0.40±0.14 abc	**0.61±0.09 b**	0.37±0.09	0.02±0.01	0.00±0.00	**77.50±32.81a**	**1.64±0.64 a**
TN1	0.90±0.04 ab	0.45±0.04	0.50±0.04 abc	0.47±0.07 a	0.34±0.06	0.15±0.03	0.04±0.01	337.33±64.17 abc	10.64±1.80 ab
F-rice line	2.053*		2.410**	1.8228*	0.772ns	1.349ns	1.537ns	4.435***	3.856***
F-covariate			27.717***						

1: Plant weight loss is calculated as (weight of control plants – weight of infested plants)/weight of control plants

2: Numbers are means ± SEM; lowercase letters indicate homogenous groups based on Tukey tests (P > 0.05); results indicated in bold font are significantly less affected by planthoppers than the susceptible check TN1 based on Duncan's many-to-one tests (P > 0.05)

3: DF = 15,80 or 15,79 where the covariate plant weight had a significant effect; *** = P ≤ 0.005, * = P ≤ 0.05, ns = P ≥ 0.05; - = no test due to insufficient variability; (N = 6)

Table S8: Adult longevity on 16 rice lines according to the Survival$_{20.5f}$ test. The Survival$_{20.5f}$ test is a performance test that corresponds to the MSST$_{20.4n}$ test.

Rice line	Plant weight (mg)(control)[2]	Plant weight (mg)(infested)[2]	Plant weight loss (g)	Time to 50% dead (days)	Time to 100% dead (days)
Rathu Heenati	1.82±0.10 ^{ab}	1.61±0.15	0.11±0.08 ^{ab}	**8.17±2.91 ^{a}**	**14.17±3.00 ^{a}**
IR62	2.24±0.12 ^{b}	2.03±0.18	0.09±0.06 ^{ab}	16.00±3.00 ^{abc}	21.50±1.45 ^{b}
PTB33	1.87±0.12 ^{ab}	1.89±0.10	-0.03±0.07 ^{a}	18.83±0.83 ^{bc}	23.00±1.26 ^{b}
IR65482-4	2.15±0.13 ^{ab}	2.00±0.10	0.05±0.07 ^{ab}	23.17±0.17 ^{c}	26.50±0.56 ^{b}
Triveni	1.65±0.23 ^{ab}	1.47±0.11	0.02±0.14 ^{ab}	18.17±1.19 ^{bc}	23.67±1.58 ^{b}
IR46	2.16±0.06 ^{ab}	1.67±0.16	0.22±0.08 ^{ab}	21.17±0.79 ^{bc}	25.00±1.37 ^{b}
Mudgo	1.99±0.05 ^{ab}	1.68±0.10	0.15±0.06 ^{ab}	19.00±1.24 ^{bc}	26.83±1.11 ^{b}
IR65482-7	1.92±0.15 ^{ab}	1.67±0.14	0.11±0.08 ^{ab}	19.83±1.72 ^{bc}	26.17±1.05 ^{b}
IR40	1.85±0.08 ^{ab}	1.21±0.19	0.35±0.10 ^{ab}	20.33±0.92 ^{bc}	24.17±0.48 ^{b}
Utri Rajapan	1.84±0.19 ^{ab}	1.62±0.16	0.04±0.18 ^{ab}	20.83±1.08 ^{bc}	23.83±1.35 ^{b}
TKM6	1.54±0.19 ^{a}	1.30±0.22	0.05±0.25 ^{ab}	18.17±1.35 ^{bc}	25.33±1.20 ^{b}
ASD7	1.90±0.10 ^{ab}	1.96±0.07	-0.05±0.08 ^{a}	20.00±1.51 ^{bc}	25.83±1.74 ^{b}
Asominori	2.14±0.14 ^{ab}	1.55±0.21	0.27±0.09 ^{ab}	20.33±1.78 ^{bc}	23.00±1.13 ^{b}
IR22	2.19±0.13 ^{ab}	1.86±0.17	0.15±0.07 ^{ab}	20.00±2.16 ^{bc}	28.17±0.60 ^{b}
T65	1.66±0.09 ^{ab}	0.68±0.16	0.56±0.13 ^{b}	20.00±1.03 ^{bc}	27.67±0.61 ^{b}
TN1	1.90±0.20 ^{ab}	1.41±0.19	0.19±0.17 ^{ab}	14.83±1.96 ^{abc}	23.30±2.11 ^{b}
F-rice line[3]	2.237*		3.346***	4.396***	5.246***
F-covariate[3]			38.076***		

1: Plant weight loss is calculated as (weight of control plants – weight of infested plants)/weight of control plants

2: Numbers are means ± SEM; lowercase letters indicate homogenous groups based on Tukey tests ($P > 0.05$); results indicated in bold font are significantly less affected by planthoppers than the susceptible check TN1 based on Duncan's many-to-one tests ($P > 0.05$)

3: DF = 15,80 or 15,79 where the covariate plant weight had a significant effect; *** = $P \leq 0.005$, * = $P \leq 0.05$; ($N = 6$)

Table S9: Honeydew production on 16 rice lines based on the Honeydew$_{20.2ḟ}$ test. The honeydew test is a performance test designed to assess relative feeding capacities of planthoppers on different rice varieties/lines

Rice line	Total area of honeydew (mm^2)	Total number of honeydew spots	Area of phloem-derived honeydew (mm^2)	Total phloem-derived spots	Area of xylem-derived honeydew (mm^2)	Total xylem-derived spots	Xylem honeydew as a proportion of total
Rathu Heenati	**0.49±0.09** ^{a}	18.17±4.13 ^{ab}	**0.22±0.08** ^{a}	3.67±1.41	0.28±0.10 ^{abc}	14.50±5.28 ^{abc}	**0.52±0.18** ^{ab}
IR62	**0.47±0.04** ^{a}	16.33±2.56 ^{ab}	**0.23±0.06** ^{a}	4.67±1.48	0.24±0.05 ^{abc}	11.67±2.95 ^{abc}	**0.52±0.13** ^{ab}
PTB33	**0.60±0.03** ^{a}	21.67±4.26 ^{ab}	**0.16±0.06** ^{a}	2.17±0.87	**0.44±0.06** ^{bc}	19.50±3.69 ^{c}	**0.74±0.10** ^{b}
IR65482-4	**0.33±0.06** ^{a}	10.17±1.70 ^{ab}	**0.16±0.05** ^{a}	3.67±1.05	0.17±0.07 ^{abc}	6.50±2.33 ^{ab}	**0.45±0.14** ^{ab}
Triveni	**1.21±0.19** ^{a}	19.83±3.05 ^{ab}	**0.81±0.23** ^{a}	4.67±1.28	**0.40±0.10** ^{bc}	15.17±2.87 ^{abc}	0.38±0.10 ^{ab}
IR46	**1.59±0.45** ^{ab}	11.00±1.46 ^{ab}	**1.45±0.47** ^{ab}	5.33±1.86	0.14±0.03 ^{abc}	5.67±0.99 ^{a}	0.12±0.03 ^{a}
Mudgo	2.33±0.72 ^{ab}	16.00±3.76 ^{ab}	2.15±0.68 ^{ab}	5.33±1.56	0.18±0.06 ^{abc}	10.67±3.81 ^{abc}	0.08±0.02 ^{a}
IR65482-7	**1.33±0.41** ^{ab}	7.00±1.29 ^{a}	**1.27±0.41** ^{ab}	3.67±0.61	0.06±0.02 ^{a}	3.33±1.52 ^{a}	0.05±0.02 ^{a}
IR40	2.09±0.53 ^{ab}	13.67±3.56 ^{ab}	1.93±0.55 ^{ab}	3.83±0.70	0.16±0.05 ^{abc}	9.83±3.42 ^{abc}	0.11±0.04 ^{a}
Utri Rajapan	**1.42±0.32** ^{ab}	11.33±1.09 ^{ab}	**1.26±0.31** ^{ab}	4.50±0.56	0.17±0.02 ^{abc}	6.83±0.87 ^{ab}	0.14±0.02 ^{a}
TKM6	**0.94±0.08** ^{a}	18.00±3.97 ^{ab}	**0.48±0.18** ^{a}	3.50±1.50	**0.46±0.17** ^{c}	14.50±4.71 ^{abc}	**0.49±0.17** ^{ab}
ASD7	1.81±0.27 ^{ab}	19.00±3.42 ^{ab}	**1.64±0.30** ^{ab}	6.50±1.57	0.17±0.03 ^{abc}	12.50±3.08 ^{abc}	0.12±0.03 ^{a}
Asominori	**1.51±0.29** ^{ab}	24.17±5.76 ^{b}	**1.32±0.27** ^{ab}	6.50±1.59	0.19±0.03 ^{abc}	17.67±5.74 ^{bc}	0.13±0.01 ^{a}
IR22	2.11±0.64 ^{ab}	13.33±4.22 ^{ab}	2.01±0.64 ^{ab}	3.17±0.70	0.10±0.03 ^{ab}	10.17±4.59 ^{abc}	0.08±0.02 ^{a}
T65	2.35±0.74 ^{ab}	13.00±3.14 ^{ab}	2.20±0.75 ^{ab}	4.67±1.02	0.15±0.04 ^{abc}	8.33±2.46 ^{abc}	0.10±0.03 ^{a}
TN1	3.32±0.56 ^{b}	8.33±3.03 ^{ab}	3.21±0.56 ^{b}	3.17±0.31	0.11±0.04 ^{ab}	5.17±2.83 ^{a}	0.04±0.02 ^{a}
F-rice line	3.965***	2.093*	4.588***	0.969ns	3.203***	1.806*	4.673***

1: Numbers are means ± SEM; lowercase letters indicate homogenous groups based on Tukey tests ($P > 0.05$); results indicated in bold font are significantly less affected by planthoppers than the susceptible check TN1 based on Duncan's many-to-one tests ($P > 0.05$)
2: DF = 15,80; *** = $P \leq 0.005$, * = $P \leq 0.05$ (N = 6)

Table S10: Results from Oviposition$_{20.2f/m}$ test on 16 rice lines

Rice line	Batches located along midrib (proportion)	Number of egg batches	Batch size (number of eggs)	Total number of eggs	Number of nymphs	Unhatched-fertile eggs (proportion)	Unhatched-infertile eggs (proportion)	Proportion of eggs that were unhatched
Rathu Heenati	0.00±0.00 ^{a}	**3.83±2.01** ^{a}	39.10±14.97	**86.67±43.18** ^{a}	**69.83±27.88** ^{a}	0.03±0.03	0.00±0.00 ^{a}	0.03±0.03 ^{a}
IR62	0.00±0.00 ^{a}	**5.50±2.92** ^{a}	19.75±2.98	**100.17±55.32** ^{ab}	**92.67±50.43** ^{a}	0.01±0.01	0.00±0.00 ^{a}	0.01±0.01 ^{a}
PTB33	0.00±0.00 ^{a}	**4.67±2.72** ^{a}	32.97±8.13	**115.33±50.40** ^{ab}	**95.50±37.02** ^{a}	0.01±0.01	0.02±0.02 ^{ab}	0.04±0.03 ^{a}
IR65482-4	0.01±0.01 ^{a}	11.00±3.06 ^{ab}	28.96±5.88	262.67±64.34 ^{abcd}	230.00±59.07 ^{ab}	0.05±0.03	0.01±0.01 ^{ab}	0.06±0.03 ^{ab}
Triveni	0.00±0.00 ^{a}	7.83±1.60 ^{ab}	42.26±9.29	270.00±48.24 ^{abcd}	259.17±51.81 ^{ab}	0.02±0.01	0.01±0.01 ^{ab}	0.02±0.01 ^{a}
IR46	0.00±0.00 ^{a}	18.17±9.12 ^{ab}	41.98±12.05	350.83±60.01 ^{abcd}	283.50±15.15 ^{ab}	0.03±0.02	0.03±0.02 ^{ab}	0.06±0.04 ^{ab}
Mudgo	0.10±0.10 ^{a}	18.83±6.67 ^{ab}	27.83±8.59	329.67±68.47 ^{abcd}	217.67±41.51 ^{ab}	0.09±0.05	0.03±0.02 ^{abcd}	0.12±0.05 ^{abc}
IR65482-7	0.08±0.04 ^{b}	14.50±1.43 ^{ab}	21.01±2.97	291.83±32.44 ^{abcd}	251.67±24.18 ^{ab}	0.03±0.02	0.02±0.01 ^{abcd}	0.06±0.02 ^{ab}
IR40	0.01±0.01 ^{a}	17.50±2.49 ^{ab}	23.75±4.37	370.67±34.39 ^{cd}	339.17±30.39 ^{b}	0.02±0.01	0.02±0.02 ^{abcd}	0.04±0.02 ^{ab}
Utri Rajapan	0.00±0.00 ^{a}	8.83±2.52 ^{ab}	46.57±29.33	**187.67±52.02** ^{abc}	**159.17±40.22** ^{ab}	0.05±0.03	0.00±0.00 ^{a}	0.05±0.03 ^{ab}
TKM6	0.00±0.00 ^{a}	22.00±4.23 ^{ab}	12.71±2.14	**243.00±29.03** ^{abcd}	**219.40±23.99** ^{ab}	0.03±0.01	0.01±0.01 ^{abcd}	0.05±0.01 ^{ab}
ASD7	0.14±0.06 ^{b}	15.33±2.87 ^{ab}	18.99±3.01	**266.83±45.15** ^{abcd}	**164.17±30.60** ^{ab}	0.09±0.04	0.08±0.04 ^{bcd}	0.17±0.04 ^{bcd}
Asominori	0.00±0.00 ^{a}	21.50±4.26 ^{ab}	13.19±1.36	**266.83±45.37** ^{abcd}	**94.17±34.28** ^{a}	0.09±0.03	0.25±0.05 ^{d}	**0.35±0.05** ^{d}
IR22	0.06±0.04 ^{ab}	22.17±5.09 ^{ab}	30.96±13.93	388.17±58.86 ^{cd}	346.50±55.58 ^{b}	0.01±0.01	0.04±0.02 ^{abcd}	0.05±0.02 ^{ab}
T65	0.05±0.04 ^{ab}	28.50±5.39 ^{b}	17.82±1.47	496.17±100.07 ^{cd}	**200.33±51.87** ^{ab}	0.08±0.02	0.18±0.05 ^{cd}	**0.26±0.06** ^{cd}
TN1	0.05±0.03 ^{ab}	21.83±3.18 ^{ab}	22.17±3.04	442.00±33.14 ^{d}	359.00±33.06 ^{b}	0.04±0.01	0.04±0.02 ^{abcd}	0.08±0.03 ^{ab}
F-line	2.787***	2.845***	1.040ns	5.058***	5.285***	1.686ns	5.203***	4.526***

1: Numbers are means ± SEM; lowercase letters indicate homogenous groups based on Tukey tests ($P > 0.05$); results indicated in bold font are significantly less affected by planthoppers than the susceptible check TN1 based on Duncan's many-to-one tests ($P > 0.05$)

2: DF = 15,80 or 15,79 where the covariate plant weight had a significant effect; *** = $P \leq 0.005$, ns = $P > 0.05$; (N = 6)

Table S11: Results from Days to Wilt ($DTW_{30.25n}$) test indicating responses by planthoppers to 16 rice lines

Rice line	Number of planthoppers	Development (proportion at each life stage)						Proportion of adults that were female	Pooled dry weight of planthoppers (mg)
		1st instar	2nd instar	3rd instar	4th instar	5th instar	Adult		
Rathu Heenati	**147.50±82.81 ^{ab}**	**0.10±0.07 ^{a}**	**0.03±0.02 ^{ab}**	0.07±0.04 ^{a}	0.08±0.04	0.10±0.03	**0.62±0.12 ^{d}**	0.64±0.09 ^{d}	54.85±21.08 ^{ab}
IR62	**109.30±78.70 ^{a}**	**0.31±0.23 ^{ab}**	**0.06±0.03 ^{abc}**	0.07±0.05 ^{a}	0.20±0.11	0.09±0.05	0.28±0.12 ^{abc}	0.70±0.15 ^{abc}	69.05±54.12 ^{ab}
PTB33	**26.60±14.75 ^{a}**	**0.20±0.16 ^{ab}**	**0.01±0.01 ^{a}**	0.10±0.06 ^{ab}	0.11±0.05	0.11±0.06	**0.47±0.18 ^{cd}**	0.48±0.21 ^{cd}	**14.75±7.00 ^{a}**
IR65482-4	**835.30±257.48 ^{ab}**	0.51±0.10 ^{b}	**0.21±0.04 ^{de}**	0.12±0.03 ^{abc}	0.06±0.03	0.05±0.03	0.05±0.02 ^{a}	0.54±0.12 ^{a}	72.44±17.44 ^{ab}
Triveni	**377.36±75.45 ^{ab}**	**0.11±0.07 ^{a}**	**0.08±0.03 ^{abcd}**	0.09±0.01 ^{ab}	0.13±0.04	0.14±0.03	**0.45±0.08 ^{bcd}**	0.53±0.06 ^{bcd}	88.03±29.78 ^{ab}
IR46	**510.30±120.45 ^{ab}**	**0.13±0.04 ^{a}**	**0.10±0.03 ^{abcde}**	0.13±0.03 ^{abc}	0.14±0.03	0.16±0.03	**0.34±0.09 ^{abcd}**	0.44±0.04 ^{abcd}	83.52±17.94 ^{ab}
Mudgo	**764.50±149.07 ^{ab}**	0.11±0.04 ^{a}	0.15±0.03 ^{abcde}	0.22±0.03 ^{bc}	0.17±0.02	0.15±0.02	0.20±0.06 ^{ab}	0.45±0.06 ^{abc}	106.84±18.23 ^{ab}
IR65482-7	**847.10±221.37 ^{ab}**	0.10±0.04 ^{a}	0.15±0.03 ^{abcde}	0.22±0.02 ^{bc}	0.22±0.03	0.18±0.03	0.13±0.04 ^{ab}	0.31±0.06 ^{ab}	96.02±23.20 ^{ab}
IR40	**1040.63±343.07 ^{bc}**	0.27±0.05 ^{ab}	0.24±0.02 ^{e}	0.22±0.02 ^{bc}	0.14±0.03	0.10±0.02	0.03±0.02 ^{a}	0.35±0.15 ^{a}	64.26±16.90 ^{ab}
Utri Rajapan	**879.00±229.42 ^{ab}**	**0.12±0.05 ^{a}**	0.13±0.04 ^{abcde}	0.20±0.02 ^{abc}	0.18±0.03	0.18±0.03	0.19±0.06 ^{ab}	0.45±0.08 ^{abc}	99.63±14.22 ^{b}
TKM6	**540.40±133.26 ^{ab}**	0.16±0.04 ^{ab}	0.17±0.03 ^{bcde}	0.21±0.02 ^{bc}	0.16±0.02	0.13±0.02	0.16±0.05 ^{ab}	0.37±0.08 ^{abc}	63.88±13.15 ^{ab}
ASD7	**900.50±258.83 ^{ab}**	0.26±0.08 ^{ab}	0.20±0.03 ^{cde}	0.21±0.03 ^{bc}	0.12±0.02	0.11±0.02	0.11±0.05 ^{ab}	0.43±0.07 ^{a}	83.23±20.84 ^{ab}
Asominori	**447.50±94.30 ^{ab}**	0.28±0.05 ^{ab}	0.22±0.02 ^{de}	0.19±0.02 ^{abc}	0.18±0.03	0.12±0.02	0.02±0.01 ^{a}	0.30±0.11 ^{a}	**24.35±4.32 ^{a}**
IR22	**500.60±144.19 ^{ab}**	0.17±0.05 ^{ab}	0.21±0.02 ^{de}	0.24±0.02 ^{c}	0.17±0.02	0.14±0.02	0.07±0.02 ^{a}	0.38±0.07 ^{a}	42.40±7.64 ^{ab}
T65	**445.20±148.58 ^{ab}**	0.18±0.05 ^{ab}	0.20±0.03 ^{cde}	0.21±0.02 ^{bc}	0.21±0.04	0.14±0.04	0.06±0.03 ^{a}	0.35±0.11 ^{a}	**30.82±8.12 ^{a}**
TN1	1871.40±266.57 ^{c}	0.27±0.04 ^{ab}	0.25±0.02 ^{e}	0.24±0.01 ^{c}	0.13±0.02	0.08±0.02	0.03±0.02 ^{a}	0.43±0.09 ^{a}	100.46±13.18 ^{b}
F-value	5.807***	2.211**	5.772***	4.931***	1.718*	1.407ns	6.772***	1.120ns	2.441***

1: Numbers are means ± SEM; lowercase letters indicate homogenous groups based on Tukey tests; results indicated in bold font are significantly less affected by planthoppers than the susceptible check TN1 based on Duncan's many-to-one tests

2: DF = 15,144; *** = P ≤ 0.005, *8 = P ≤ 0.01, * = P ≤ 0.05, ns = P > 0.05 (N = 10)

Table S12: Results from Days to Wilt ($DTW_{30.25n}$) test indicating responses by 16 rice lines to infestation by planthoppers

Rice line	Plant weight (control g)	Plant weight loss (g)	Plant weight loss (proportion)	Weight of filled grain (control g)	Weight of unfilled grain (control g)	DTW (days)	Damage rating (at 60 DAI)	Plant yield loss (proportion)	DTW (days)	Damage rating (at 60 DAI)
Rathu Heenati	27.69±2.91 ^{def}	**0.23±0.35 ^{a}**	**0.05±0.06 ^{a}**	0.64±0.43	0.23±0.15	**>60.00 ^{e}**	**3.00±0.00 ^{a}**	0.23±0.19	**>60.00 ^{e}**	**3.00±0.00 ^{a}**
IR62	14.87±1.97 ^{abc}	**1.13±0.75 ^{a}**	**0.10±0.03 ^{a}**	7.99±0.66 ^{cd}	1.06±0.20 ^{abc}	**>60.00 ^{e}**	**3.40±0.27 ^{a}**	**0.10±0.08 ^{a}**	**>60.00 ^{e}**	**3.40±0.27 ^{a}**
PTB33	33.44±2.91 ^{f}	**0.59±0.59 ^{a}**	**0.10±0.06 ^{a}**	0.59±0.59	0.01±0.01	**>60.00 ^{e}**	**3.40±0.27 ^{a}**	0.33±0.33	**>60.00 ^{e}**	**3.40±0.27 ^{a}**
IR65482-4	12.99±1.35 ^{ab}	**2.42±1.78 ^{ab}**	**0.07±0.13 ^{a}**	7.67±0.77 ^{d}	0.69±0.19 ^{ab}	**>60.00 ^{e}**	**5.80±0.33 ^{b}**	**0.18±0.20 ^{a}**	**>60.00 ^{e}**	**5.80±0.33 ^{b}**
Triveni	9.03±0.87 ^{a}	6.80±1.20 ^{bcd}	**0.10±0.12 ^{a}**	7.98±0.54 ^{cd}	0.50±0.15 ^{a}	**56.30±1.56 ^{e}**	**7.80±0.33 ^{cd}**	0.81±0.14 ^{b}	**56.30±1.56 ^{e}**	**7.80±0.33 ^{cd}**
IR46	17.98±1.70 ^{abcde}	5.04±1.01 ^{abcd}	0.57±0.05 ^{b}	5.04±1.01 ^{abcd}	1.27±0.31 ^{abc}	**54.40±1.99 ^{de}**	**7.20±0.63 ^{c}**	1.00±0.00 ^{b}	**54.40±1.99 ^{de}**	**7.20±0.63 ^{c}**
Mudgo	24.86±3.95 ^{bcdef}	**3.72±0.65 ^{abc}**	0.56±0.09 $_{b}$	3.72±0.65 ^{a}	1.50±0.32 ^{bc}	45.30±1.14 ^{bc}	9.00±0.00 ^{e}	1.00±0.00 ^{b}	45.30±1.14 ^{bc}	9.00±0.00 ^{e}
IR65482-7	13.86±1.26 ^{ab}	8.70±1.07 ^{d}	0.56±0.04 ^{b}	8.70±1.07 ^{bcd}	0.31±0.08 ^{a}	52.40±2.47 ^{cde}	8.20±0.33 ^{cde}	1.00±0.00 ^{b}	52.40±2.47 ^{cde}	8.20±0.33 ^{cde}
IR40	16.82±2.09 ^{abcd}	8.77±0.82 ^{d}	0.66±0.04 ^{b}	8.77±0.82 ^{d}	0.81±0.28 ^{abc}	38.10±2.50 ^{ab}	9.00±0.00 ^{e}	1.00±0.00 ^{b}	38.10±2.50 ^{ab}	9.00±0.00 ^{e}
Utri Rajapan	29.33±2.63 ^{ef}	**3.73±1.02 ^{abc}**	0.72±0.03 ^{b}	3.73±1.02 ^{a}	1.28±0.30 ^{abc}	47.40±3.02 ^{cd}	8.60±0.27 ^{de}	1.00±0.00 ^{b}	47.40±3.02 ^{cd}	8.60±0.27 ^{de}
TKM6	19.77±3.30 ^{abcde}	**4.28±0.74 ^{abcd}**	0.62±0.05 ^{b}	4.28±0.74 ^{abc}	1.17±0.42 ^{abc}	45.20±2.30 ^{bc}	9.00±0.00 ^{e}	1.00±0.00 ^{b}	45.20±2.30 ^{bc}	9.00±0.00 ^{e}
ASD7	26.16±3.95 ^{cdef}	**4.18±0.69 ^{abc}**	0.67±0.06 ^{b}	4.19±0.68 ^{ab}	1.68±0.31 ^{c}	45.90±1.91 ^{bcd}	9.00±0.00 ^{e}	0.99±0.01 ^{b}	45.90±1.91 ^{bcd}	9.00±0.00 ^{e}
Asominori	9.86±1.38 ^{a}	6.09±0.72 ^{bcd}	0.50±0.05 ^{b}	6.57±0.75 ^{abcd}	0.38±0.11 ^{a}	35.80±0.51 ^{a}	9.00±0.00 ^{e}	0.92±0.02 ^{b}	35.80±0.51 ^{a}	9.00±0.00 ^{e}
IR22	18.90±2.80 ^{abcde}	7.11±0.71 ^{cd}	0.70±0.04 ^{b}	7.11±0.71 ^{abcd}	0.86±0.24 ^{abc}	45.60±1.89 ^{bcd}	9.00±0.00 ^{e}	1.00±0.00 ^{b}	45.60±1.89 ^{bcd}	9.00±0.00 ^{e}
T65	15.74±2.30 ^{abcd}	4.56±0.51 ^{abcd}	0.66±0.04 ^{b}	4.56±0.51 ^{abc}	0.35±0.13 ^{a}	45.30±2.54 ^{bc}	9.00±0.00 ^{e}	1.00±0.00 ^{b}	45.30±2.54 ^{bc}	9.00±0.00 ^{e}
TN1	12.20±1.84 ^{a}	7.87±0.65 ^{cd}	0.55±0.06 ^{b}	7.87±0.65 ^{bcd}	1.19±0.60 ^{abc}	34.70±1.86 ^{a}	9.00±0.00 ^{e}	1.00±0.00 ^{b}	34.70±1.86 ^{a}	9.00±0.00 ^{e}
F-rice line	8.574***	6.178***	15.932***	6.317***	2.300**	23.485***	87.982***	16.521***	23.485***	87.982***

1: Plant weight loss is calculated as absolute weight loss 'weight of control plants – weight of infested plants' and as a proportion '(weight of control plants – weight of infested plants)/weight of control plants' and yield loss as (yield of control plants – yield of infested plants)/yield of control plants.

2: Numbers are means ± SEM; lowercase letters indicate homogenous groups based on Tukey tests ($P > 0.05$); results indicated in bold font are significantly less affected by planthoppers than the susceptible check TN1 based on Duncan's many-to-one tests ($P > 0.05$)

3: DF = 15,144 or 13,126 for yield comparisons (due to photo-period effect on grain production in Rathu Heenati and PTB33); *** = $P \leq 0.005$, ** = $P \leq 0.01$; (N = 10)

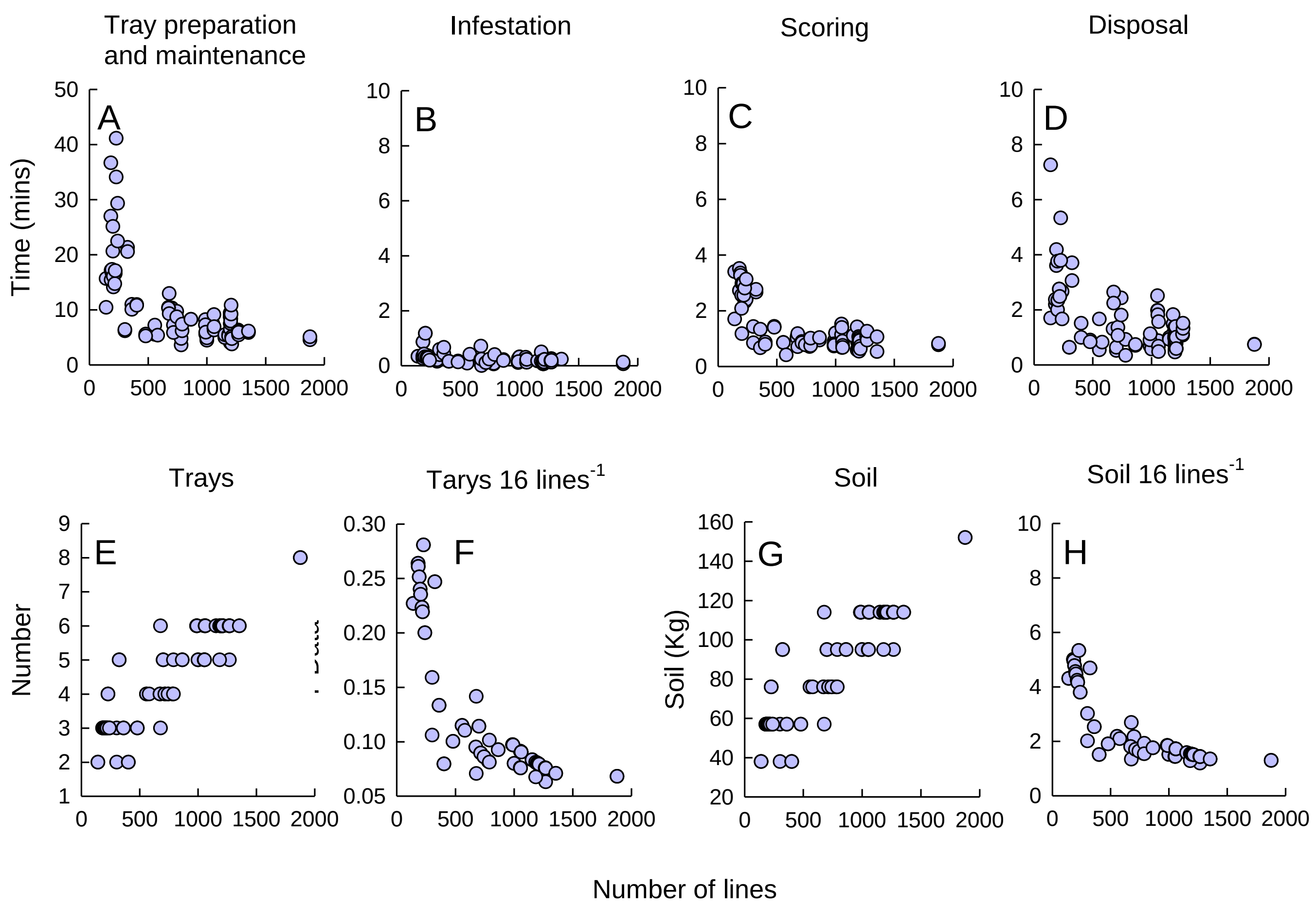

Figure S1: Time to process 16 rice lines during routine SSST with brown planthopper at IRRI. Graphs indicate the time (A) to prepare trays, (B) to infest the trays with planthoppers, (C) to score rice lines at the time that susceptible checks wilt and (D) for disposal of material after the SSST has been completed. Times are dependent on the number of lines evaluated during routine phenotyping (i.e., batch size/number of lines on x-axis). The number of trays required to test lines is indicated in (E) with the number of trays per 16 lines in (F). The amount of soil required for screening is presented in (G), with soil per 16 lines in (H).

Table S13: Estimated costs and time for processing materials during 11 phenotyping tests (see Figure 6). Details of each of the phenotyping tests are presented in Table 1

Test	Scoring using SES				Added times for assessment of resistance			Added costs and times for assessment of plant tolerance				
	Materials costs ($)[1]	Space (m^2 days)[2]	Set-up time (mins)[4]	Evaluation time (mins)[5]	Counts (mins)[3]	Development (mins)[3]	Weighing (mins)[3]	Materials costs ($)[1]	Space (m^2 days)[2]	Set-up time (mins)[4]	Plant evaluation (mins)[3]	Grain sorting and weighing (mins)[3]
SSST$_{7.8n}$	60.00	253.75	24.5	22.50±0.43	-	-	-	60.00	253.75	22.00	126.00±4.47	-
MSST$_{7.2n}$	60.00	380.63	24.5	19.67±0.52	-	-	-	60.00	380.63	22.00	121.67±3.56	-
MSST$_{20.4n}$	60.00	444.06	24.5	20.26±0.23	-	-	-	60.00	444.06	22.00	83.33±2.51	-
Survival$_{7.8n}$	12.00	37.80	15.39±4.11	5.22±0.25	12.00±1.41	49.50±3.88	21.00±4.38	12.00	37.80	13.33	46.33±4.89	-
Survival$_{20.4n}$	14.40	147.00	15.39±4.11	5.22±0.25	19.83±1.33	45.67±2.51	23.17±1.54	14.40	147.00	13.33	60.00±4.22	-
Survival$_{20.5f}$	14.40	147.00	15.39±4.11	5.22±0.25	102.67±1.86			14.40	147.00	13.33	62.67±6.64	-
Build-up$_{7.2f}$	12.00	51.45	15.39±4.11	12.00±1.41	483.00±48.18[6]		48.17±7.18	12.00	51.45	13.33	59.33±5.79	-
Build-up$_{20.4n}$	14.40	147.00	15.39±4.11	12.00±1.41	520.83±26.20[6]		48.00±8.63	14.40	147.00	13.33	56.67±3.99	-
Oviposition$_{20.2f/m}$	14.40	108.00	15.39±4.11	-	285.17±21.48[6]		-	-	-	-	-	-
Honeydew$_{20.2f}$	8.00	14.40	93.33±5.67	-	111.67±3.65	-	-	-	-	-	-	-
DTW$_{30.25n}$	26.00	2592.00	33.39±8.11	11.80±1.41	3268.68±323.30[6]		48.00±8.63	26.00	2592.00	26.67	60.45±2.58	34.50±0.72

1: Materials costs were estimated for 1 replicate of 16 varieties based on costs in the Philippines

2: Space requirements are estimated based on base area of arenas for 16 varieties over the duration of the test

3: Based on 6-10 replicates

4: Based on a single replicate with 16 varieties

5: Times for scoring were evaluated once for Survival tests and once for DTW and Build-up tests (N = 6-10)

6: Times for counting, sorting and assessment of development stages were not separated
